# Unconventional Applications of Semaglutide and Tirzepatide: From Oncology to Human Reproduction

**DOI:** 10.3390/biomedicines14071644

**Published:** 2026-07-21

**Authors:** Sandro La Vignera, Rosita A. Condorelli

**Affiliations:** Department of Clinical and Experimental Medicine, University of Catania, 95123 Catania, Italy

**Keywords:** semaglutide, tirzepatide, GLP-1 receptor agonist, cancer, addiction, osteoarthritis, polycystic ovary syndrome, incretin therapy

## Abstract

Semaglutide and tirzepatide, glucagon-like peptide-1 (GLP-1) and dual glucose-dependent insulinotropic polypeptide (GIP)/GLP-1 receptor agonists, respectively, have revolutionized the management of type 2 diabetes mellitus and obesity. Beyond their established metabolic indications, emerging preclinical and clinical evidence suggests these incretin-based therapies exert pleiotropic effects across multiple organ systems through mechanisms extending beyond glycemic control and weight reduction. This comprehensive review synthesizes current evidence for unconventional applications of semaglutide and tirzepatide across five distinct therapeutic domains: oncology, psychiatry and addiction medicine, orthopedics, aesthetic medicine, and human reproduction. In oncology, both agents demonstrate antitumor activity primarily through immune and metabolic reprogramming rather than direct cytotoxicity, with promising signals in pancreatic, thyroid, breast, and colorectal cancers. In psychiatry, modulation of mesolimbic dopamine reward pathways and GABAergic neurotransmission underlies robust preclinical and observational evidence for reducing alcohol use disorder, substance use, and binge-eating behaviors. Orthopedic applications include clinically meaningful improvements in knee osteoarthritis pain and function, though bone health signals remain mixed. Aesthetic medicine faces the dual challenge of managing GLP-1 receptor agonist-associated facial volume loss while exploring therapeutic potential in inflammatory dermatoses. In reproductive medicine, metabolic improvements translate to benefits in polycystic ovary syndrome and male fertility, though periconception safety concerns persist. Across all domains, evidence derives predominantly from preclinical models, observational cohorts, and pharmacoepidemiologic studies, with randomized controlled trials remaining limited. This review critically evaluates mechanisms, efficacy signals, safety considerations, and research priorities for each application domain, providing a roadmap for translating unconventional uses of semaglutide and tirzepatide into evidence-based clinical practice.

## 1. Methods

This narrative review was conducted following a systematic search of PubMed/MEDLINE, Scopus, Web of Science, and ClinicalTrials.gov databases. Search terms included combinations of: “semaglutide,” “tirzepatide,” “GLP-1 receptor agonist,” “GIP receptor agonist,” “incretin therapy,” combined with “cancer,” “oncology,” “addiction,” “alcohol use disorder,” “substance use,” “osteoarthritis,” “bone health,” “aesthetic medicine,” “dermatology,” “polycystic ovary syndrome,” “fertility,” “reproduction,” and “pregnancy.” The search covered publications from January 2000 to May 2026. Inclusion criteria encompassed original research articles, systematic reviews, meta-analyses, randomized controlled trials, observational studies, and case series reporting on non-metabolic applications of semaglutide or tirzepatide. Preclinical studies (in vitro and animal models) were included when mechanistic data were relevant. Editorials, letters without original data, and non-peer-reviewed sources were excluded. Evidence quality was assessed using a modified Oxford Centre for Evidence-Based Medicine framework, categorizing findings as Level 1 (RCTs/meta-analyses), Level 2 (prospective cohort studies), Level 3 (retrospective/observational studies), Level 4 (preclinical/mechanistic studies), and Level 5 (case reports/expert opinion). Given the predominantly exploratory nature of the evidence base, no formal PRISMA flow was generated; however, the methodological transparency described above allows reproducibility of the search strategy (Table 1).

## 2. Introduction

### 2.1. GLP-1 and GIP Receptor Agonist Pharmacology

Glucagon-like peptide-1 (GLP-1) and glucose-dependent insulinotropic polypeptide (GIP) are incretin hormones secreted by enteroendocrine cells in response to nutrient ingestion. These hormones regulate glucose homeostasis through multiple mechanisms, including enhancement of glucose-dependent insulin secretion, suppression of glucagon release, delayed gastric emptying, and modulation of appetite and satiety signaling in the central nervous system [1,2]. Semaglutide is a long-acting GLP-1 receptor agonist with 94% homology to native human GLP-1, modified to resist dipeptidyl peptidase-4 degradation and prolong half-life through albumin binding [3]. Tirzepatide represents a novel dual GIP/GLP-1 receptor agonist, designed to harness complementary and potentially synergistic effects of both incretin pathways [4].

A critical pharmacological distinction between semaglutide and tirzepatide that has implications across all unconventional applications is the dual receptor agonism of tirzepatide. While semaglutide acts exclusively on GLP-1 receptors, tirzepatide’s additional GIP receptor agonism may confer distinct effects on adipose tissue distribution, bone metabolism, neurological function, and potentially oncogenic pathways. The GIP receptor is expressed in bone, brain, adipose tissue, and gastrointestinal epithelium, and GIP receptor agonism has been associated with adipose tissue remodeling and bone formation in preclinical models. However, the specific contribution of GIP receptor agonism to tirzepatide’s effects in unconventional applications has not been systematically characterized, and current evidence does not allow delineation of GLP-1-mediated versus GIP-mediated effects in most clinical contexts. This represents a fundamental knowledge gap that future mechanistic and clinical studies must address.

The pharmacokinetic profiles of these agents enable once-weekly subcutaneous administration, with semaglutide exhibiting a half-life of approximately 1 week and tirzepatide approximately 5 days [5,6]. Both agents achieve steady-state concentrations after 4–5 weeks of dosing. Semaglutide is approved at doses up to 2.4 mg weekly for chronic weight management and 1.0 mg weekly for type 2 diabetes mellitus, while tirzepatide is approved at doses up to 15 mg weekly for both indications [7,8].

GLP-1 and GIP receptors belong to the class B G protein-coupled receptor family and are expressed not only in pancreatic islets but also in diverse tissues including the central nervous system, cardiovascular system, gastrointestinal tract, kidneys, and immune cells [9,10]. This widespread receptor distribution provides the mechanistic basis for pleiotropic effects beyond glucose regulation. Upon receptor binding, both GLP-1 and GIP activate adenylyl cyclase through Gαs protein coupling, increasing intracellular cyclic adenosine monophosphate (cAMP) and activating downstream signaling cascades involving protein kinase A (PKA) and exchange protein directly activated by cAMP (EPAC) [11]. These pathways regulate diverse cellular processes including gene transcription, cell proliferation, apoptosis, inflammation, and oxidative stress responses [12].

This review addresses a topic that has attracted recent attention in the literature. While some overlap exists with previously published narrative reviews on GLP-1 receptor agonist applications (e.g., [13]), the present manuscript provides an updated synthesis incorporating the most recent evidence through May 2026, with particular emphasis on evidence-level stratification, agent-specific differentiation between semaglutide and tirzepatide, and a comprehensive safety evaluation in non-metabolic applications. The added value of this review lies in its systematic evidence grading, its explicit delineation of which findings are drug-class effects versus agent-specific, and its critical appraisal of the limitations and translational barriers in each domain.

### 2.2. Rationale for Exploring Unconventional Applications

The recognition that GLP-1 and GIP receptor agonists exert effects beyond their primary metabolic targets has emerged from multiple lines of evidence. First, cardiovascular outcome trials of GLP-1 receptor agonists demonstrated reductions in major adverse cardiovascular events that appeared to exceed what would be predicted from glycemic control and weight loss alone, suggesting direct vascular and cardiac effects [14,15]. Second, preclinical studies revealed GLP-1 receptor expression and functional signaling in tissues not traditionally associated with glucose metabolism, including neurons, immune cells, and various cancer cell lines [16,17]. Third, observational studies and pharmacoepidemiologic analyses have reported associations between GLP-1 receptor agonist use and reduced incidence of diverse conditions including Alzheimer’s disease, alcohol use disorder, and certain malignancies [18,19,20].

These observations have catalyzed investigation of semaglutide and tirzepatide for applications far removed from their original indications. However, the evidence base for these unconventional uses varies dramatically in quality and quantity across different domains. Some applications, such as knee osteoarthritis, are supported by randomized controlled trials, while others rest primarily on preclinical mechanistic studies or retrospective observational data subject to substantial confounding. This review aims to provide a comprehensive, critical synthesis of current evidence for unconventional applications of semaglutide and tirzepatide, explicitly addressing the strength of evidence, mechanistic plausibility, safety considerations, and research priorities for each domain.

### 2.3. Scope and Organization

This review is organized into five major therapeutic domains: (1) oncology, (2) psychiatry and addiction medicine, (3) orthopedics and bone health, (4) aesthetic medicine and dermatology, and (5) reproductive medicine. For each domain, we systematically address: (a) mechanistic rationale based on receptor expression and signaling pathways; (b) preclinical evidence from in vitro and animal studies; (c) clinical evidence from observational studies and randomized trials; (d) safety considerations specific to that application; and (e) future research directions. We conclude with an integrated discussion of cross-cutting themes, including the challenge of distinguishing direct pharmacological effects from consequences of weight loss, the need for agent-specific evidence comparing semaglutide and tirzepatide, and the ethical and practical considerations surrounding off-label use of these agents for unconventional indications.

## 3. Oncology Applications

### 3.1. Mechanistic Basis for Antitumor Effects

The potential antitumor effects of GLP-1 receptor agonists are supported by multiple mechanistic pathways that extend beyond their metabolic actions. GLP-1 receptors are expressed in various human cancer cell lines and tumor tissues, including pancreatic, thyroid, breast, colorectal, and prostate cancers, though expression levels vary considerably across tumor types and individual patients [21,22,23]. Activation of GLP-1 receptors in cancer cells can modulate multiple signaling pathways relevant to tumorigenesis, including the phosphatidylinositol 3-kinase (PI3K)/protein kinase B (AKT)/mammalian target of rapamycin (mTOR) pathway, mitogen-activated protein kinase (MAPK) cascades, and nuclear factor kappa B (NF-κB) inflammatory signaling [24,25].

A central mechanism underlying the antitumor effects of GLP-1 receptor agonists involves metabolic reprogramming of the tumor microenvironment. Cancer cells characteristically exhibit altered metabolism, including increased glycolysis (the Warburg effect), enhanced glutamine metabolism, and dysregulated lipid synthesis [26]. GLP-1 receptor activation can counteract these metabolic alterations by enhancing oxidative phosphorylation, reducing lactate production, and modulating adenosine monophosphate-activated protein kinase (AMPK) signaling [27,28]. These metabolic shifts can impair cancer cell proliferation and survival, particularly in tumors heavily dependent on glycolytic metabolism.

Immune modulation represents another important mechanism. GLP-1 receptor agonists can enhance antitumor immunity by promoting M1 macrophage polarization, increasing natural killer cell activity, and reducing immunosuppressive regulatory T cell populations in the tumor microenvironment [29,30]. Additionally, these agents reduce systemic and local inflammation through suppression of pro-inflammatory cytokines including tumor necrosis factor-alpha (TNF-α), interleukin-6 (IL-6), and interleukin-1 beta (IL-1β), which can contribute to cancer progression [31,32].

GLP-1 receptor agonists also modulate angiogenesis, a critical process for tumor growth and metastasis. These agents can reduce vascular endothelial growth factor (VEGF) expression and inhibit endothelial cell proliferation and migration, potentially limiting tumor vascularization [33,34]. Furthermore, GLP-1 receptor activation can induce apoptosis in cancer cells through both intrinsic (mitochondrial) and extrinsic (death receptor) pathways, involving activation of caspase cascades and modulation of B-cell lymphoma 2 (BCL-2) family proteins [35,36].

Importantly, the antitumor effects of GLP-1 receptor agonists appear to be mediated primarily through indirect mechanisms—metabolic normalization, immune enhancement, and microenvironment modulation—rather than direct cytotoxic effects on cancer cells. This mechanistic profile suggests potential utility in cancer prevention and as adjunctive therapy rather than as primary antineoplastic agents.

It is important to emphasize that the majority of antitumor evidence derives from preclinical models, including in vitro cell lines and murine xenograft experiments, where GLP-1 receptor agonists are often administered at supraphysiological concentrations that may not be achievable in clinical practice. The translational relevance of these findings to human oncology therefore remains uncertain. GLP-1 receptor expression varies considerably across tumor types and individual patients, and the biological significance of receptor expression in human malignancies has not been established. Extrapolation of murine findings to human cancer biology must be approached with caution given fundamental differences in tumor microenvironment, immune landscape, and receptor pharmacology between species.

Furthermore, many observational studies reporting reduced cancer incidence or mortality in patients receiving GLP-1 receptor agonists are subject to confounding by indication: patients prescribed these agents tend to have better metabolic health, higher socioeconomic status, and greater access to preventive healthcare compared with untreated controls. It cannot be excluded that the apparent benefits in observational studies primarily reflect the consequences of weight loss and metabolic improvement rather than direct anticancer drug effects. Conflicting data exist specifically for pancreatic cancer, where some preclinical models suggest promotional effects, underscoring the need for prospective randomized evidence before any oncologic application can be considered. Semaglutide and tirzepatide do not currently have an established role in oncology, and their use for cancer prevention or treatment outside of clinical trials is not supported by current evidence.

**Figure 1:**
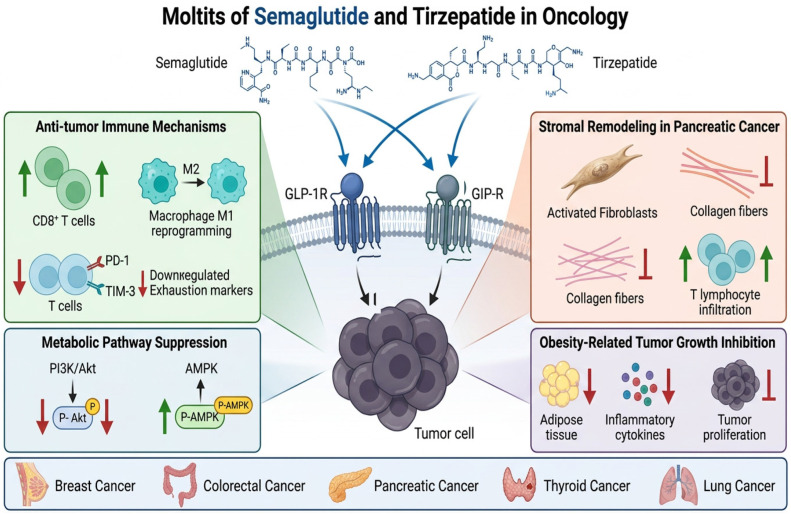
Mechanisms of semaglutide and tirzepatide in oncology. GLP-1 and dual GIP/GLP-1 receptor agonists modulate tumour immune microenvironment, metabolic reprogramming, angiogenesis, and apoptotic pathways. Note: GLP-1 and GIP receptors belong to the class B G protein-coupled receptor family and possess seven transmembrane domains; the schematic representation is simplified for illustrative purposes. The blue colour coding of semaglutide denotes the GLP-1 receptor agonist component to distinguish it from tirzepatide’s dual agonist structure.

Regarding the distinction between agents, most mechanistic oncology data derive from studies using liraglutide or exenatide, or from generic GLP-1 receptor agonist class effects. Direct evidence specifically attributable to semaglutide is limited, and clinical evidence for tirzepatide in oncology is virtually absent. Conclusions regarding tirzepatide’s antitumor potential are at present entirely inferential, based on its shared GLP-1 receptor agonism and its additional GIP receptor activity, the oncologic implications of which are unknown (Figure 1).

### 3.2. Pancreatic Cancer

Pancreatic ductal adenocarcinoma (PDAC) represents one of the most lethal malignancies, with a 5-year survival rate below 10% [37]. The relationship between GLP-1 receptor agonists and pancreatic cancer has been controversial, with conflicting preclinical and epidemiological evidence.

Early concerns about pancreatic safety of incretin-based therapies emerged from case reports of pancreatitis and preclinical studies suggesting that GLP-1 receptor activation might promote pancreatic ductal cell proliferation [38,39]. However, subsequent large-scale cardiovascular outcome trials and meta-analyses have not demonstrated increased pancreatic cancer risk with GLP-1 receptor agonists [40,41]. The SUSTAIN and PIONEER trials of semaglutide reported no signal for increased pancreatic malignancy [15,42].

Paradoxically, emerging evidence suggests potential antitumor effects of GLP-1 receptor agonists in established pancreatic cancer. GLP-1 receptors are expressed in human PDAC tissue and cell lines, and receptor activation can inhibit cancer cell proliferation, induce apoptosis, and reduce migration and invasion in vitro [24,43]. In murine xenograft models, treatment with GLP-1 receptor agonists including liraglutide and exenatide reduced tumor growth and metastasis through mechanisms involving AMPK activation, mTOR inhibition, and modulation of epithelial–mesenchymal transition [43].

The tumor microenvironment in PDAC is characterized by extensive desmoplastic stroma, immunosuppression, and metabolic reprogramming. GLP-1 receptor agonists may counteract these features by reducing pancreatic stellate cell activation, enhancing immune cell infiltration, and normalizing tumor metabolism [17,44]. Additionally, these agents may improve the efficacy of chemotherapy by enhancing drug delivery through modulation of tumor vasculature and interstitial pressure [45].

A retrospective cohort study of patients with type 2 diabetes and pancreatic cancer found that those treated with GLP-1 receptor agonists had improved overall survival compared with other glucose-lowering therapies, even after adjustment for confounders [46]. However, this finding requires validation in prospective studies specifically designed to evaluate oncologic outcomes.

For tirzepatide, no specific data on pancreatic cancer exist. The dual GIP/GLP-1 receptor agonism may have distinct effects on pancreatic tissue compared with selective GLP-1 receptor agonists, but this remains entirely speculative.

### 3.3. Thyroid Cancer

Thyroid C-cells express GLP-1 receptors, and preclinical studies in rodents demonstrated that GLP-1 receptor agonists can induce C-cell hyperplasia and medullary thyroid carcinoma (MTC) [47]. This finding led to contraindication of GLP-1 receptor agonists in patients with personal or family history of MTC or multiple endocrine neoplasia type 2 [48]. However, the relevance of rodent C-cell findings to human thyroid cancer risk remains uncertain, as human C-cells express substantially lower levels of GLP-1 receptors compared with rodent C-cells [49].

Large-scale pharmacovigilance studies and clinical trials have not demonstrated increased incidence of thyroid cancer with GLP-1 receptor agonists in humans [50,51]. The SUSTAIN and PIONEER trials of semaglutide reported no cases of MTC [15,42]. A comprehensive meta-analysis including over 60,000 patients treated with GLP-1 receptor agonists found no significant increase in thyroid cancer risk compared with placebo or active comparators [52].

Interestingly, emerging evidence suggests potential therapeutic effects of GLP-1 receptor agonists in established thyroid cancers. GLP-1 receptors are expressed in human papillary thyroid carcinoma (PTC) and follicular thyroid carcinoma (FTC) tissues [53]. In vitro studies demonstrated that GLP-1 receptor activation inhibits proliferation and migration of PTC cells [54].

A retrospective analysis of patients with differentiated thyroid cancer and concurrent type 2 diabetes found that those treated with GLP-1 receptor agonists had lower rates of disease recurrence and improved progression-free survival compared with patients receiving other glucose-lowering therapies [55]. These findings suggest potential utility of GLP-1 receptor agonists as adjunctive therapy in thyroid cancer, though prospective validation is required.

For semaglutide specifically, post-marketing surveillance has not identified safety signals for thyroid malignancy in humans [56]. Tirzepatide carries the same precautionary labeling regarding MTC based on rodent data, but human evidence is lacking.

### 3.4. Breast Cancer

Breast cancer is the most common malignancy in women worldwide, and obesity is an established risk factor for postmenopausal breast cancer and worse outcomes across all breast cancer subtypes [57,58]. GLP-1 receptors are expressed in human breast cancer tissues and cell lines, with expression levels varying across molecular subtypes [22,59].

Preclinical studies have demonstrated antitumor effects of GLP-1 receptor agonists in breast cancer models. In estrogen receptor-positive (ER+) breast cancer cell lines, GLP-1 receptor activation inhibited proliferation, induced apoptosis, and reduced estrogen-stimulated growth through modulation of estrogen receptor signaling and downstream pathways including PI3K/AKT and MAPK [25,60]. In triple-negative breast cancer (TNBC) models, GLP-1 receptor agonists reduced cell viability, migration, and invasion, and enhanced sensitivity to chemotherapy [61,62].

In murine xenograft models, treatment with liraglutide or exenatide reduced breast tumor growth and metastasis through multiple mechanisms including enhanced antitumor immunity, reduced angiogenesis, and metabolic reprogramming of the tumor microenvironment [63,64]. Importantly, these effects were observed independently of weight loss, suggesting direct antitumor actions.

Epidemiological evidence supports potential protective effects of GLP-1 receptor agonists against breast cancer. A large retrospective cohort study of women with type 2 diabetes found that GLP-1 receptor agonist use was associated with reduced breast cancer incidence compared with other glucose-lowering therapies (adjusted hazard ratio 0.68, 95% confidence interval 0.52–0.89) [65]. Another study reported improved overall survival in women with breast cancer and diabetes who were treated with GLP-1 receptor agonists [66].

The mechanisms underlying these potential benefits likely involve both direct effects on breast cancer cells and indirect effects through weight loss, metabolic improvement, and reduction in obesity-associated inflammation and insulin resistance. Obesity promotes breast cancer through multiple pathways including hyperinsulinemia, increased bioavailable estrogen, chronic inflammation, and adipokine dysregulation [62]. By addressing these obesity-related mechanisms, GLP-1 receptor agonists may reduce breast cancer risk and improve outcomes.

Specific evidence for semaglutide in breast cancer is limited to preclinical studies and subgroup analyses of clinical trials. Tirzepatide has not been specifically studied in breast cancer, though its superior weight loss efficacy compared with semaglutide might theoretically confer greater benefits through obesity reduction.

### 3.5. Colorectal Cancer

Colorectal cancer (CRC) is the third most common malignancy worldwide, and obesity is a well-established risk factor for both CRC incidence and mortality [67,68]. GLP-1 receptors are expressed in normal colonic epithelium and in CRC tissues, with expression levels varying across tumor stages and molecular subtypes [23].

Preclinical studies have demonstrated antitumor effects of GLP-1 receptor agonists in CRC models. In human CRC cell lines, GLP-1 receptor activation inhibited proliferation, induced apoptosis, and reduced migration and invasion through mechanisms involving AMPK activation, mTOR inhibition, and modulation of Wnt/β-catenin signaling [17,69]. In murine models of colitis-associated cancer and chemically induced CRC, treatment with GLP-1 receptor agonists reduced tumor incidence, multiplicity, and size [17,70].

The antitumor effects in CRC models appear to involve multiple mechanisms. GLP-1 receptor agonists reduce intestinal inflammation, a key driver of colitis-associated cancer, through suppression of pro-inflammatory cytokines and modulation of gut microbiota composition [71,72]. These agents also enhance intestinal barrier function and reduce bacterial translocation, which can contribute to CRC development [23]. Additionally, GLP-1 receptor agonists also exert direct antiproliferative and pro-apoptotic effects on colorectal cancer cells and suppress tumor angiogenesis, further contributing to their antitumor activity in preclinical models [17,69,70].

Epidemiological evidence supports protective effects of GLP-1 receptor agonists against CRC. A large population-based cohort study found that GLP-1 receptor agonist use was associated with reduced CRC incidence in patients with type 2 diabetes (adjusted hazard ratio 0.56, 95% confidence interval 0.38–0.83) [73]. Another study reported improved overall survival in patients with CRC and diabetes who were treated with GLP-1 receptor agonists compared with other glucose-lowering therapies [74].

A meta-analysis of observational studies including over 1 million patients with diabetes found that GLP-1 receptor agonist use was associated with a 40% reduction in CRC risk compared with other antidiabetic medications [75]. However, these observational studies are subject to confounding by indication, as patients prescribed GLP-1 receptor agonists may differ systematically from those receiving other treatments in ways that independently affect cancer risk.

The mechanisms underlying the apparent protective effects likely involve both direct actions on colonic epithelium and indirect effects through weight loss, improved glycemic control, and reduced systemic inflammation. Hyperinsulinemia and insulin resistance, both improved by GLP-1 receptor agonists, are established risk factors for CRC [35].

Specific evidence for semaglutide in CRC is limited, with most data deriving from studies of liraglutide or class effects of GLP-1 receptor agonists. Tirzepatide has not been specifically studied in CRC, though its metabolic effects might theoretically confer similar or greater benefits.

### 3.6. Other Malignancies

Emerging evidence suggests potential effects of GLP-1 receptor agonists in other malignancies, though data are more limited. In prostate cancer, GLP-1 receptors are expressed in both normal prostate tissue and prostate cancer cell lines [43]. Preclinical studies demonstrated that GLP-1 receptor activation inhibits prostate cancer cell proliferation and induces apoptosis through modulation of androgen receptor signaling and downstream pathways [35,76]. Observational studies have reported associations between GLP-1 receptor agonist use and reduced prostate cancer incidence in men with diabetes [77].

In hepatocellular carcinoma (HCC), GLP-1 receptor agonists may exert protective effects through improvement of non-alcoholic fatty liver disease (NAFLD) and non-alcoholic steatohepatitis (NASH), which are major risk factors for HCC [78,79]. Preclinical studies demonstrated that GLP-1 receptor agonists reduce hepatic steatosis, inflammation, and fibrosis, and inhibit HCC development in murine models [46,79]. Clinical trials have shown that semaglutide improves histological features of NASH, potentially reducing progression to cirrhosis and HCC [63].

In lung cancer, GLP-1 receptors are expressed in both normal lung tissue and non-small cell lung cancer (NSCLC) cell lines [60]. Preclinical studies showed that GLP-1 receptor activation inhibits NSCLC cell proliferation, induces apoptosis, and reduces metastasis through modulation of EGFR and PI3K/AKT signaling [60,63]. However, clinical evidence is lacking.

In ovarian cancer, GLP-1 receptor expression has been detected in ovarian cancer tissues and cell lines [63]. Preclinical studies demonstrated antiproliferative and pro-apoptotic effects of GLP-1 receptor agonists in ovarian cancer models [60]. Interestingly, GLP-1 receptor agonists may enhance the efficacy of platinum-based chemotherapy in ovarian cancer through modulation of drug resistance mechanisms [80].

### 3.7. Clinical Implications and Future Directions

The accumulating evidence for potential antitumor effects of GLP-1 receptor agonists raises important questions about their role in cancer prevention and treatment. However, several critical gaps must be addressed before these agents can be recommended for oncologic applications.

First, the vast majority of mechanistic and efficacy data derive from preclinical models, and translation to human cancer remains uncertain. The concentrations of GLP-1 receptor agonists used in many in vitro studies exceed clinically achievable levels, and murine cancer models may not accurately reflect human tumor biology. Second, observational studies suggesting reduced cancer incidence with GLP-1 receptor agonists are subject to substantial confounding, and it remains unclear whether apparent benefits reflect direct antitumor effects or consequences of weight loss and metabolic improvement. Third, the optimal dosing, duration, and timing of GLP-1 receptor agonist therapy for potential oncologic benefits are unknown. Fourth, potential interactions between GLP-1 receptor agonists and standard cancer therapies require systematic investigation.

Prospective randomized controlled trials are needed to definitively establish whether GLP-1 receptor agonists reduce cancer incidence or improve outcomes in patients with established malignancies. Such trials should include mechanistic substudies to elucidate whether effects are mediated through direct actions on tumor cells, modulation of the tumor microenvironment, or indirect metabolic effects. Biomarker studies are needed to identify which patients and tumor types are most likely to benefit from GLP-1 receptor agonist therapy.

For semaglutide and tirzepatide specifically, dedicated oncology trials are warranted given their superior efficacy for weight loss compared with earlier GLP-1 receptor agonists. The dual GIP/GLP-1 receptor agonism of tirzepatide may confer distinct oncologic effects compared with selective GLP-1 receptor agonists, but this remains entirely speculative and requires direct investigation.

## 4. Psychiatry and Addiction Medicine

### 4.1. Neurobiological Mechanisms

The potential therapeutic effects of GLP-1 receptor agonists in psychiatry and addiction medicine are grounded in the widespread expression of GLP-1 receptors throughout the central nervous system, particularly in brain regions involved in reward processing, motivation, and emotional regulation [16,81]. GLP-1 receptors are densely expressed in the nucleus accumbens, ventral tegmental area, hippocampus, amygdala, hypothalamus, and prefrontal cortex [80,82].

A central mechanism underlying the effects of GLP-1 receptor agonists on addictive behaviors involves modulation of the mesolimbic dopamine reward pathway. This pathway, projecting from the ventral tegmental area to the nucleus accumbens, mediates the rewarding effects of drugs of abuse, alcohol, and palatable foods [83]. GLP-1 receptor activation in the ventral tegmental area reduces dopamine neuron firing and dopamine release in the nucleus accumbens, thereby attenuating reward signaling [83,84]. This mechanism has been demonstrated for multiple substances including alcohol, cocaine, amphetamine, nicotine, and opioids in preclinical models [85].

GLP-1 receptor agonists also modulate GABAergic neurotransmission, which plays a critical role in addiction neurobiology. These agents enhance GABAergic inhibitory tone in reward-related brain regions, counteracting the disinhibition that characterizes addictive states [85]. Additionally, GLP-1 receptor activation modulates glutamatergic neurotransmission and synaptic plasticity in the nucleus accumbens and prefrontal cortex, regions critical for habit formation and executive control [85].

Beyond reward pathways, GLP-1 receptor agonists exert effects on stress response systems implicated in addiction. These agents modulate hypothalamic–pituitary–adrenal (HPA) axis function and reduce stress-induced reinstatement of drug-seeking behavior in animal models [85]. GLP-1 receptor activation also influences neuroinflammation, which has been implicated in the pathophysiology of addiction and psychiatric disorders [86].

Importantly, GLP-1 receptor agonists cross the blood–brain barrier, though the extent of central nervous system penetration varies across agents [87]. Semaglutide, with its albumin-binding modification, has limited blood–brain barrier penetration, and its central effects may be mediated primarily through peripheral GLP-1 receptors that signal to the brain via vagal afferents and circumventricular organs [88]. Tirzepatide’s blood–brain barrier penetration has not been extensively characterized.

It must be clearly stated that the role of incretin-based therapies in addiction medicine remains entirely investigational. The substantial majority of mechanistic evidence derives from rodent models, and the applicability of mesolimbic reward pathway modulation observed in animals to human addiction neurobiology cannot be assumed. Available human evidence consists predominantly of small pilot trials, retrospective analyses, and pharmacoepidemiologic database studies, all of which are subject to significant methodological limitations including selection bias, confounding, reverse causality, and publication bias favoring positive results. No large-scale randomized controlled trial has yet demonstrated definitive clinical efficacy of semaglutide or tirzepatide for any addiction indication. These findings should not be interpreted as evidence of established clinical efficacy, and off-label use for addiction treatment is premature pending results of ongoing prospective trials.

**Figure 2:**
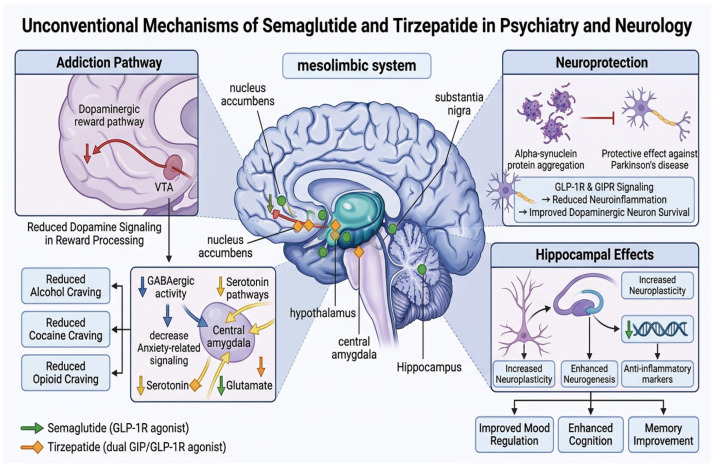
Neurobiological mechanisms of semaglutide and tirzepatide in psychiatry and addiction medicine. GLP-1 and dual GIP/GLP-1 receptor agonists modulate mesolimbic dopamine reward pathways, GABAergic neurotransmission, and stress-response systems implicated in substance use and binge-eating disorders. Note: GLP-1 and GIP receptors possess seven transmembrane domains; the schematic representation is simplified for illustrative purposes.

Regarding agent-specific evidence: most human data on addiction come from studies of exenatide or liraglutide, with semaglutide data emerging more recently. Tirzepatide-specific data in addiction medicine are essentially absent, and extrapolation from GLP-1 receptor agonist class effects is speculative given the additional GIP receptor component of tirzepatide (Figure 2).

### 4.2. Alcohol Use Disorder

Alcohol use disorder (AUD) affects over 14 million adults in the United States and is associated with substantial morbidity, mortality, and societal costs [89]. Current pharmacological treatments for AUD have limited efficacy, and novel therapeutic approaches are urgently needed [83].

Preclinical evidence strongly supports a role for GLP-1 receptor agonists in reducing alcohol consumption. In rodent models, systemic or central administration of GLP-1 receptor agonists including exenatide, liraglutide, and semaglutide reduced voluntary alcohol intake, alcohol-seeking behavior, and alcohol-induced dopamine release in the nucleus accumbens [83,84,90]. These effects were observed across multiple paradigms including two-bottle choice, operant self-administration, and binge-like drinking models [84]. Importantly, GLP-1 receptor agonists reduced alcohol consumption without affecting water intake or causing general malaise, suggesting specific effects on alcohol reward rather than non-specific suppression of consummatory behavior [83].

The mechanisms underlying these effects involve modulation of mesolimbic dopamine signaling and GABAergic neurotransmission in reward-related brain regions. GLP-1 receptor activation in the ventral tegmental area reduces the excitability of dopamine neurons that project to the nucleus accumbens, thereby attenuating the rewarding effects of alcohol [83]. Additionally, GLP-1 receptor agonists reduce alcohol-induced neuroinflammation and oxidative stress, which contribute to alcohol-related brain damage [19].

Human evidence for GLP-1 receptor agonists in AUD is emerging. A retrospective cohort study of patients with type 2 diabetes and AUD found that those treated with GLP-1 receptor agonists had significantly lower rates of alcohol-related hospitalizations and emergency department visits compared with patients receiving other glucose-lowering therapies [91]. A pharmacoepidemiologic study using a large healthcare database reported that GLP-1 receptor agonist use was associated with reduced incidence of AUD diagnosis and alcohol-related complications [92].

Small pilot trials have provided preliminary evidence of efficacy. A randomized, placebo-controlled trial of exenatide in individuals with AUD and obesity found that exenatide treatment reduced alcohol consumption, alcohol craving, and binge drinking episodes over 26 weeks [92]. Another pilot study of liraglutide in individuals with AUD reported reductions in alcohol intake and improvements in liver function markers [92].

For semaglutide specifically, case reports and case series have described marked reductions in alcohol consumption and craving in patients treated for obesity or diabetes [92]. A retrospective analysis of patients receiving semaglutide for weight management found that those with comorbid AUD reported substantial reductions in alcohol use [93]. However, prospective randomized trials of semaglutide specifically for AUD are lacking.

Tirzepatide has not been studied in AUD, and its effects on alcohol consumption are unknown. The dual GIP/GLP-1 receptor agonism may confer distinct effects on reward pathways compared with selective GLP-1 receptor agonists, but this is entirely speculative.

Several ongoing clinical trials are evaluating GLP-1 receptor agonists for AUD, including a phase 2 trial of semaglutide (NCT05891587) and trials of exenatide and liraglutide [93]. These studies will provide critical evidence regarding efficacy, optimal dosing, and safety in this population.

### 4.3. Substance Use Disorders

Beyond alcohol, preclinical evidence suggests that GLP-1 receptor agonists may reduce use of multiple substances of abuse, including cocaine, amphetamine, nicotine, opioids, and cannabis [90,92].

For cocaine, rodent studies demonstrated that GLP-1 receptor agonists reduce cocaine self-administration, cocaine-seeking behavior, and cocaine-induced locomotor sensitization [94]. These effects involve modulation of dopamine signaling in the nucleus accumbens and prefrontal cortex. GLP-1 receptor agonists also reduce cocaine-induced reinstatement of drug-seeking behavior triggered by stress, drug-associated cues, or cocaine priming [92].

For amphetamine and methamphetamine, preclinical studies showed that GLP-1 receptor agonists reduce drug self-administration, conditioned place preference, and psychomotor sensitization [92]. These agents also attenuate amphetamine-induced neurotoxicity through reduction in oxidative stress and neuroinflammation [92].

For nicotine, GLP-1 receptor agonists reduce nicotine self-administration, nicotine-induced dopamine release, and nicotine withdrawal symptoms in rodent models [92]. A small clinical trial of exenatide in smokers with type 2 diabetes found that exenatide treatment was associated with increased smoking cessation rates compared with placebo [92].

For opioids, preclinical evidence is more limited but suggests that GLP-1 receptor agonists may reduce opioid reward and self-administration [92]. Interestingly, GLP-1 receptor agonists may also reduce opioid-induced respiratory depression, a major cause of opioid overdose mortality [19].

Human evidence for GLP-1 receptor agonists in substance use disorders other than alcohol is sparse. A retrospective cohort study found that patients with type 2 diabetes treated with GLP-1 receptor agonists had lower rates of cannabis use disorder compared with those receiving other glucose-lowering therapies [92]. Case reports have described reductions in cocaine and methamphetamine use in patients treated with GLP-1 receptor agonists for metabolic indications [92].

For semaglutide, anecdotal reports and social media accounts describe reductions in various substance use behaviors, but systematic evidence is lacking [95]. Tirzepatide has not been studied in substance use disorders.

### 4.4. Binge-Eating Disorder and Food Addiction

Binge-eating disorder (BED) is the most common eating disorder, characterized by recurrent episodes of consuming large amounts of food with a sense of loss of control [96]. The concept of “food addiction,” while controversial, describes a pattern of compulsive eating of highly palatable foods that shares neurobiological features with substance use disorders [97].

GLP-1 receptor agonists are mechanistically well-positioned to address binge-eating and food addiction through their effects on appetite, satiety, and reward processing. These agents reduce food intake through multiple mechanisms including delayed gastric emptying, enhanced satiety signaling, and reduced reward value of palatable foods [92,98].

Preclinical studies demonstrated that GLP-1 receptor agonists reduce binge-like eating of palatable foods in rodent models [99]. These effects involve modulation of dopamine signaling in the nucleus accumbens and reduction in the hedonic value of food rewards [99]. Importantly, GLP-1 receptor agonists reduce food intake without causing conditioned taste aversion, suggesting specific effects on reward processing rather than induction of malaise [100].

Clinical evidence supports efficacy of GLP-1 receptor agonists in BED. A randomized, placebo-controlled trial of liraglutide in individuals with BED and obesity found that liraglutide significantly reduced binge-eating episodes, improved eating disorder psychopathology, and produced substantial weight loss [92]. Another study reported that exenatide reduced binge-eating frequency and severity in individuals with BED [7].

For semaglutide, post hoc analyses of weight management trials found that semaglutide treatment was associated with reductions in binge-eating behaviors and improvements in eating-related quality of life [98]. Patients treated with semaglutide commonly report reduced food cravings, decreased preoccupation with food, and diminished reward value of previously preferred foods [8].

Tirzepatide has not been specifically studied in BED, though its superior weight loss efficacy compared with semaglutide suggests potentially greater effects on eating behaviors. Anecdotal reports describe marked reductions in binge eating and food cravings with tirzepatide treatment [98].

The effects of GLP-1 receptor agonists on eating behaviors extend beyond BED to include reductions in emotional eating, night eating, and grazing behaviors [101]. These agents may be particularly beneficial for individuals with obesity and comorbid eating disorders or food addiction phenotypes.

### 4.5. Other Psychiatric Applications

Emerging evidence suggests potential applications of GLP-1 receptor agonists in other psychiatric conditions, though data are more preliminary.

In depression, preclinical studies demonstrated antidepressant-like effects of GLP-1 receptor agonists in rodent models of depression [101]. These effects involve modulation of hippocampal neurogenesis, brain-derived neurotrophic factor (BDNF) expression, and inflammatory signaling [102]. Small clinical studies have reported improvements in depressive symptoms in patients with diabetes treated with GLP-1 receptor agonists [101]. However, dedicated trials in major depressive disorder are lacking.

In anxiety disorders, preclinical evidence suggests anxiolytic effects of GLP-1 receptor agonists through modulation of GABAergic neurotransmission and HPA axis function [102]. Clinical evidence is limited to observational studies reporting reduced anxiety symptoms in patients treated with these agents for metabolic indications [18].

In cognitive disorders and dementia, GLP-1 receptor agonists have shown neuroprotective effects in preclinical models of Alzheimer’s disease and vascular dementia [18]. These agents reduce amyloid-beta accumulation, tau phosphorylation, neuroinflammation, and oxidative stress, while enhancing synaptic plasticity and neurogenesis [103]. Clinical trials of GLP-1 receptor agonists in Alzheimer’s disease and mild cognitive impairment are ongoing [104].

In schizophrenia, GLP-1 receptor agonists may address metabolic side effects of antipsychotic medications while potentially improving cognitive symptoms [104]. Small pilot studies have reported metabolic benefits and cognitive improvements in patients with schizophrenia treated with GLP-1 receptor agonists [105].

### 4.6. Clinical Implications and Future Directions

The emerging evidence for GLP-1 receptor agonists in psychiatry and addiction medicine is promising but requires substantial additional investigation before clinical recommendations can be made. Key research priorities include:

First, large-scale randomized controlled trials are needed to definitively establish efficacy for specific psychiatric and addiction indications. These trials should include diverse populations, adequate duration to assess sustained effects, and comprehensive safety monitoring.

Second, mechanistic studies are needed to elucidate whether effects are mediated through central nervous system penetration and direct receptor activation in the brain, or through peripheral mechanisms involving vagal signaling and metabolic improvement. This has implications for agent selection and dosing strategies.

Third, comparative effectiveness studies are needed to determine whether semaglutide and tirzepatide differ in their psychiatric and addiction-related effects, and how they compare with existing treatments for these conditions.

Fourth, biomarker studies are needed to identify which patients are most likely to benefit from GLP-1 receptor agonist therapy for psychiatric or addiction indications. Factors such as baseline metabolic status, genetic variants in GLP-1 receptor or dopamine signaling pathways, and neuroimaging markers may predict treatment response.

Fifth, safety considerations specific to psychiatric populations require careful evaluation, including potential interactions with psychotropic medications, effects on mood and suicidality, and adherence challenges in patients with severe mental illness.

## 5. Orthopedics and Bone Health

### 5.1. Osteoarthritis

Osteoarthritis (OA) is the most common joint disorder, affecting over 500 million people worldwide and representing a leading cause of disability [106]. Obesity is a major risk factor for knee OA, contributing through both mechanical loading and metabolic/inflammatory mechanisms [107].

The STEP-9 trial provided the first randomized controlled evidence for semaglutide in knee OA [108]. This 68-week trial enrolled 407 adults with obesity (BMI ≥ 30 kg/m^2^) and moderate knee OA. Participants randomized to semaglutide 2.4 mg weekly achieved significantly greater improvements in knee pain (measured by WOMAC pain subscale) and physical function compared with placebo. The mean difference in WOMAC pain score was −14.4 points (95% CI −18.9 to −9.9, *p* < 0.001), representing a clinically meaningful improvement. Semaglutide-treated participants also achieved greater weight loss (mean −13.7% vs. −3.2% with placebo) and improvements in inflammatory biomarkers including high-sensitivity C-reactive protein (hs-CRP).

The STEP-9 trial provides the most robust clinical evidence for semaglutide in knee osteoarthritis, demonstrating significant improvements in pain and physical function. However, it should be noted that a substantial proportion of the observed benefit—estimated at approximately 70% in mechanistic analyses—may be attributable to weight loss per se rather than to direct pharmacological effects of semaglutide on joint tissue. Whether these agents confer benefits in non-obese patients with osteoarthritis remains unknown, as clinical trials have been conducted exclusively in overweight or obese populations. For tirzepatide, no dedicated orthopedic trials exist; benefits are inferred from its superior weight loss efficacy compared with semaglutide.

The mechanisms underlying the benefits of semaglutide in OA likely involve both weight reduction and direct anti-inflammatory effects. Weight loss reduces mechanical loading on weight-bearing joints, which is particularly important in knee OA [109]. Additionally, weight loss reduces systemic inflammation and improves the metabolic profile, addressing the “metabolic OA” phenotype characterized by insulin resistance, dyslipidemia, and chronic low-grade inflammation [69].

Beyond weight loss, GLP-1 receptor agonists may exert direct effects on joint tissues. GLP-1 receptors are expressed in chondrocytes, synoviocytes, and osteoblasts [110]. In vitro studies demonstrated that GLP-1 receptor activation in chondrocytes reduces production of matrix metalloproteinases (MMPs) and pro-inflammatory cytokines, while enhancing synthesis of cartilage matrix components including type II collagen and aggrecan [110]. In animal models of OA, GLP-1 receptor agonists reduced cartilage degradation, synovial inflammation, and subchondral bone changes [110].

GLP-1 receptor agonists also modulate pain processing through central and peripheral mechanisms. These agents reduce neuroinflammation in pain-processing regions of the brain and spinal cord, and may directly modulate nociceptive signaling [106]. Additionally, improvements in mood and sleep associated with weight loss may contribute to reduced pain perception [8].

**Figure 3:**
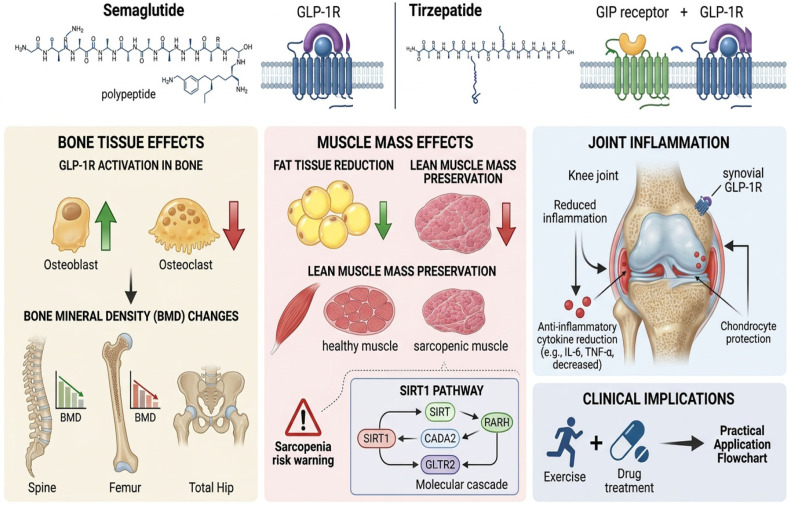
Musculoskeletal effects of semaglutide and tirzepatide. GLP-1 and dual GIP/GLP-1 receptor agonists influence bone metabolism, cartilage homeostasis, and joint inflammation through direct receptor-mediated and indirect weight-loss-dependent mechanisms. The mechanism involving SIRT1 (Sirtuin 1) deacetylase activation contributes to GLP-1 receptor agonist-mediated metabolic effects through deacetylation of PGC-1α, enhancing mitochondrial biogenesis and oxidative metabolism. Note: GLP-1 and GIP receptors possess seven transmembrane domains; the schematic representation is simplified for illustrative purposes. Protein domains depicted are schematic.

For tirzepatide, no dedicated OA trials have been published. However, given its superior weight loss efficacy compared with semaglutide (mean weight loss of 15–20% in clinical trials), tirzepatide might be expected to produce greater improvements in OA symptoms through enhanced mechanical unloading [10]. The additional GIP receptor agonism of tirzepatide may confer distinct effects on joint tissues, as GIP receptors are expressed in bone and may influence subchondral bone remodeling in OA [69]. However, these potential benefits remain speculative pending direct clinical investigation (Figure 3).

### 5.2. Bone Health

The effects of GLP-1 receptor agonists on bone health have been a subject of investigation and some controversy. Bone tissue expresses both GLP-1 and GIP receptors, and incretin hormones influence bone metabolism through multiple mechanisms [111,112].

Preclinical studies have yielded mixed results regarding bone effects of GLP-1 receptor agonists. Some studies demonstrated that GLP-1 receptor activation enhances osteoblast differentiation and bone formation while reducing osteoclast activity and bone resorption [112]. Other studies found neutral or negative effects on bone, particularly with chronic high-dose treatment [113]. These discrepancies may reflect differences in animal models, dosing regimens, and skeletal sites examined.

In humans, clinical trial data on the effects of GLP-1 receptor agonists on bone have been reassuring overall. Meta-analyses of cardiovascular and diabetes outcome trials found no significant increase in fracture risk with GLP-1 receptor agonists compared with placebo or active comparators [114,115]. Some studies reported modest reductions in bone mineral density (BMD), particularly at the hip, though the clinical significance of these small changes is uncertain [116].

For semaglutide specifically, the STEP trials included assessments of bone safety. A substudy of STEP 1 found that semaglutide 2.4 mg weekly was associated with small reductions in BMD at the hip (−1.2%) and femoral neck (−2.3%) over 68 weeks, but no change in lumbar spine BMD [115]. The reductions in hip BMD were proportional to weight loss and likely reflect reduced mechanical loading rather than direct adverse effects on bone. Importantly, no increase in fracture incidence was observed.

Bone turnover marker studies have provided additional insights. Semaglutide treatment is associated with increases in bone resorption markers (C-terminal telopeptide of type I collagen, CTX) and bone formation markers (procollagen type I N-terminal propeptide, P1NP), indicating increased bone turnover [117]. This pattern is typical of weight loss and does not necessarily indicate adverse effects on bone.

For tirzepatide, bone safety data are emerging. The SURPASS trials included fracture monitoring and found no increase in fracture risk with tirzepatide compared with placebo or active comparators [116]. A substudy evaluating BMD changes with tirzepatide found small reductions at the hip similar to those observed with semaglutide [111]. Interestingly, the dual GIP/GLP-1 receptor agonism of tirzepatide may confer bone benefits, as GIP receptor activation has been shown to enhance bone formation in preclinical studies [115]. However, clinical evidence for bone-protective effects of tirzepatide is lacking.

Several factors influence the bone effects of GLP-1 receptor agonists. First, the magnitude and rate of weight loss are important determinants of BMD changes, with rapid weight loss associated with greater bone loss [115]. Second, baseline bone health, age, and menopausal status influence susceptibility to bone loss during weight reduction [115]. Third, concurrent interventions including calcium and vitamin D supplementation, resistance exercise, and adequate protein intake may mitigate bone loss [107].

Current evidence suggests that GLP-1 receptor agonists do not significantly increase fracture risk, though modest reductions in BMD may occur, particularly at weight-bearing sites. For patients at high fracture risk (e.g., postmenopausal women with osteoporosis, elderly individuals), bone health should be monitored during treatment with GLP-1 receptor agonists, and appropriate preventive measures should be implemented.

### 5.3. Clinical Implications and Future Directions

The evidence for semaglutide in knee OA is sufficiently robust to support its use in patients with obesity and symptomatic OA, particularly when weight loss is a primary therapeutic goal. The magnitude of pain and functional improvement observed in STEP-9 is comparable to or greater than that achieved with conventional OA treatments including NSAIDs and intra-articular corticosteroids [118].

However, several questions remain. First, the durability of OA benefits beyond 68 weeks is unknown. Second, whether semaglutide provides benefits in non-obese individuals with OA requires investigation. Third, the optimal timing of semaglutide initiation in the OA disease course (early vs. established disease) is unclear. Fourth, potential synergies between semaglutide and other OA treatments (physical therapy, exercise, intra-articular therapies) warrant study.

For tirzepatide, dedicated OA trials are needed to determine whether its superior weight loss efficacy translates to greater improvements in OA outcomes compared with semaglutide. Comparative effectiveness studies would inform agent selection for patients with obesity and OA.

Regarding bone health, current evidence supports the safety of GLP-1 receptor agonists in most patients, though monitoring and preventive measures are appropriate for high-risk individuals. Future research should focus on identifying patient subgroups at increased risk for bone loss, optimizing strategies to preserve bone during weight loss, and elucidating the long-term skeletal effects of these agents.

## 6. Aesthetic Medicine and Dermatology

### 6.1. GLP-1 Receptor Agonist-Associated Facial Volume Loss

A prominent aesthetic concern associated with GLP-1 receptor agonist therapy is facial volume loss, colloquially termed “Ozempic face” [118]. This phenomenon has been widely reported in media and social media, and is increasingly recognized in clinical practice [119].

The aesthetic medicine section is largely descriptive and based on case series, clinical observations, and expert opinion, with no controlled clinical trials available. The phenomenon of GLP-1 receptor agonist-associated facial volume loss (“Ozempic face”) has been characterized descriptively but its pathophysiology has not been elucidated mechanistically. It remains unclear whether this represents preferential loss of facial adipose tissue, acceleration of age-related facial changes, or a non-specific consequence of rapid weight loss. The therapeutic potential of GLP-1 receptor agonists in inflammatory dermatoses (psoriasis, hidradenitis suppurativa) is biologically plausible given their anti-inflammatory properties, but evidence is currently limited to small uncontrolled studies and case reports. Conclusions in this domain should be considered speculative.

Facial volume loss during weight reduction is not unique to GLP-1 receptor agonists and occurs with any substantial weight loss [118]. However, the rapid and pronounced weight loss achieved with semaglutide and tirzepatide may accentuate facial changes, particularly in older adults with reduced skin elasticity [120]. Facial fat compartments, including the malar fat pads, buccal fat pad, and periorbital fat, undergo age-related atrophy, and weight loss can accelerate these changes [118].

The clinical presentation of GLP-1 receptor agonist-associated facial volume loss includes hollowing of the cheeks and temples, deepening of nasolabial folds and marionette lines, increased visibility of underlying facial structures, and an overall aged or gaunt appearance [118]. These changes can be distressing to patients and may impact treatment adherence [118].

Several factors influence the severity of facial volume loss. Older age, greater magnitude of weight loss, rapid rate of weight loss, and pre-existing facial volume depletion are associated with more pronounced changes [120]. Genetic factors influencing fat distribution and skin elasticity also play a role [118].

Management strategies for GLP-1 receptor agonist-associated facial volume loss include both preventive and corrective approaches. Preventive strategies include gradual dose escalation to slow the rate of weight loss, resistance exercise to preserve lean mass, adequate protein intake, and skincare regimens to support skin health [118]. Corrective approaches include dermal fillers (hyaluronic acid, calcium hydroxylapatite, poly-L-lactic acid) to restore facial volume, neuromodulators (botulinum toxin) to address dynamic wrinkles, and energy-based devices (radiofrequency, ultrasound) to improve skin laxity [118].

Dermal filler treatment in patients on GLP-1 receptor agonists requires special considerations. The ongoing weight loss and metabolic changes may affect filler longevity and distribution [118]. Additionally, the appetite suppression and nausea associated with GLP-1 receptor agonists may complicate post-procedure recovery [118]. Timing of filler treatment is important, with some experts recommending waiting until weight has stabilized before undertaking extensive facial rejuvenation [8].

**Figure 4:**
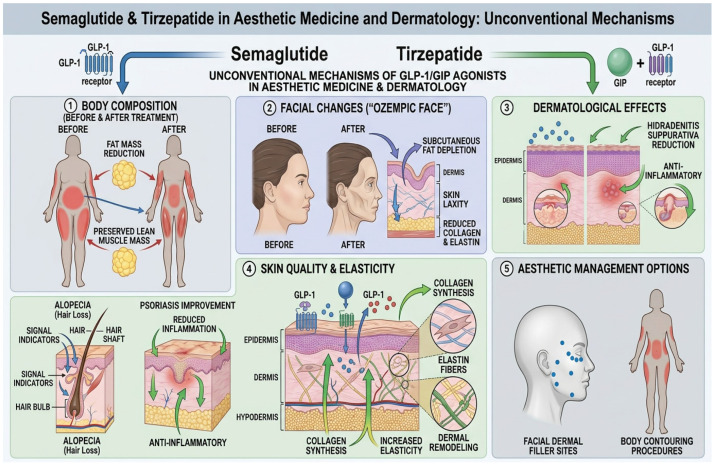
Aesthetic medicine implications of semaglutide and tirzepatide. Rapid weight loss induces body composition changes including facial volume loss (‘Ozempic face’), altered skin laxity, and shifts in adipose tissue distribution. Note: GLP-1 and GIP receptors possess seven transmembrane domains; the schematic representation is simplified for illustrative purposes. Protein domains depicted are schematic.

For tirzepatide, facial volume loss may be more pronounced than with semaglutide given the greater magnitude of weight loss achieved [121]. However, comparative data are lacking (Figure 4).

### 6.2. Dermatological Applications

Beyond aesthetic concerns, emerging evidence suggests potential therapeutic applications of GLP-1 receptor agonists in inflammatory dermatoses.

In psoriasis, obesity is a well-established risk factor and is associated with more severe disease and reduced treatment response [122]. Weight loss improves psoriasis severity and enhances efficacy of systemic therapies [60]. GLP-1 receptor agonists may benefit psoriasis through both weight reduction and direct anti-inflammatory effects. GLP-1 receptors are expressed in keratinocytes and immune cells in psoriatic skin [60]. In vitro studies demonstrated that GLP-1 receptor activation reduces keratinocyte proliferation and production of pro-inflammatory cytokines including TNF-α, IL-17, and IL-23 [60].

Case reports and small case series have described improvements in psoriasis severity in patients treated with GLP-1 receptor agonists for metabolic indications [60]. A retrospective study found that patients with psoriasis and type 2 diabetes treated with GLP-1 receptor agonists had greater reductions in Psoriasis Area and Severity Index (PASI) scores compared with those receiving other glucose-lowering therapies [123]. However, prospective controlled trials are lacking.

In hidradenitis suppurativa (HS), obesity is a major risk factor and weight loss is recommended as part of disease management [60]. GLP-1 receptor agonists may benefit HS through weight reduction and anti-inflammatory effects. Case reports have described improvements in HS severity with GLP-1 receptor agonist treatment [60]. A small retrospective study found that patients with HS treated with GLP-1 receptor agonists had reduced disease activity and fewer flares [60].

In acanthosis nigricans, a dermatological manifestation of insulin resistance, GLP-1 receptor agonists may improve skin changes through metabolic improvement. Case reports have described resolution of acanthosis nigricans with GLP-1 receptor agonist treatment [79].

In non-alcoholic fatty liver disease (NAFLD) and non-alcoholic steatohepatitis (NASH), which can manifest with cutaneous signs, GLP-1 receptor agonists have demonstrated efficacy in improving liver histology and may secondarily benefit associated skin findings [124].

### 6.3. Clinical Implications and Future Directions

The aesthetic and dermatological effects of GLP-1 receptor agonists represent an important consideration in patient counseling and treatment planning. Patients initiating these agents should be informed about the possibility of facial volume loss and strategies to mitigate this effect.

For aesthetic medicine practitioners, familiarity with GLP-1 receptor agonist-associated changes is essential for appropriate patient management. Collaboration between prescribing physicians and aesthetic practitioners can optimize outcomes and patient satisfaction.

For dermatological applications, prospective controlled trials are needed to establish the efficacy of GLP-1 receptor agonists in inflammatory skin conditions. Such trials should include mechanistic substudies to determine whether benefits are mediated through weight loss, direct anti-inflammatory effects, or both.

## 7. Reproductive Medicine

### 7.1. Polycystic Ovary Syndrome and Female Fertility

Polycystic ovary syndrome (PCOS) is the most common endocrine disorder in women of reproductive age, affecting 5–15% of this population [124]. PCOS is characterized by hyperandrogenism, ovulatory dysfunction, and polycystic ovarian morphology, and is strongly associated with obesity and insulin resistance [125]. Weight loss is a cornerstone of PCOS management, improving metabolic parameters, menstrual regularity, ovulation rates, and fertility outcomes.

GLP-1 receptor agonists represent a promising therapeutic approach for PCOS through their effects on weight reduction, insulin sensitivity, and potentially direct ovarian actions [126]. GLP-1 receptors are expressed in human ovarian tissue, including granulosa cells [127].

Clinical studies have demonstrated benefits of GLP-1 receptor agonists in women with PCOS. A randomized controlled trial comparing liraglutide with metformin in women with PCOS and obesity found that liraglutide produced greater weight loss and improvements in menstrual regularity, though both agents improved metabolic parameters [128]. Another study found that liraglutide improved ovulation rates and hormonal profiles in women with PCOS [129].

For semaglutide specifically, observational studies have reported improvements in PCOS-related outcomes. A retrospective study of women with PCOS treated with semaglutide for weight management found significant improvements in menstrual regularity (from 35% to 78% with regular cycles), reductions in total testosterone and free androgen index, and improvements in metabolic parameters including insulin resistance [129]. Another study reported that semaglutide treatment was associated with improved ovulation rates and increased spontaneous pregnancy rates in women with PCOS and infertility [125].

A critical distinction must be drawn between the metabolic benefits of weight reduction in patients with obesity-related infertility and any direct reproductive pharmacological effect of semaglutide or tirzepatide. The observed improvements in menstrual regularity, ovulation rate, and hormonal profiles in patients with polycystic ovary syndrome receiving these agents are largely consistent with the known benefits of weight loss per se, and do not necessarily indicate a drug-specific reproductive effect beyond weight reduction. Studies in non-obese patients with PCOS or infertility are lacking.

The mechanisms underlying the benefits in PCOS likely involve both weight loss and direct effects. Weight reduction improves insulin sensitivity, reduces hyperinsulinemia (which drives ovarian androgen production), and normalizes hypothalamic–pituitary–ovarian axis function. Additionally, GLP-1 receptor activation in ovarian tissue may directly modulate steroidogenesis, follicular development, and oocyte maturation [130].

Importantly, the effects of GLP-1 receptor agonists on fertility raise concerns about unintended pregnancy in women of reproductive age. The substantial improvements in ovulation and fertility that can occur with these agents necessitate counseling about contraception and pregnancy planning [131]. Given the contraindication of GLP-1 receptor agonists in pregnancy (discussed below), women should be advised to use effective contraception during treatment and to discontinue the medication at least 2 months before planned conception [131].

For tirzepatide, limited data exist regarding effects on PCOS and fertility. Case reports have described improvements in menstrual regularity and metabolic parameters in women with PCOS treated with tirzepatide [132]. Given its superior weight loss efficacy compared with semaglutide, tirzepatide might be expected to produce greater improvements in PCOS-related outcomes, but direct comparative studies are lacking.

**Figure 5:**
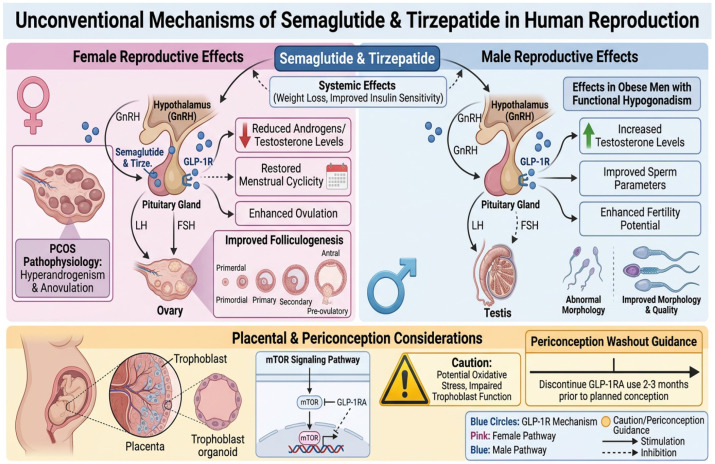
Reproductive effects of semaglutide and tirzepatide. GLP-1 and dual GIP/GLP-1 receptor agonists modulate the hypothalamic–pituitary–gonadal axis, ovarian function, and male reproductive parameters primarily through metabolic and weight-loss-dependent mechanisms, with additional direct receptor-mediated effects in gonadal tissues. Note: GLP-1 and GIP receptors possess seven transmembrane domains; the schematic representation is simplified for illustrative purposes.

Tirzepatide-specific reproductive data are virtually absent. All conclusions regarding tirzepatide’s reproductive effects are inferred from its GLP-1 receptor agonist component, with the reproductive implications of GIP receptor agonism unknown (Figure 5).

### 7.2. Male Fertility

Obesity in men is associated with reduced fertility through multiple mechanisms including hormonal alterations (reduced testosterone, increased estradiol), impaired spermatogenesis, erectile dysfunction, and reduced libido [132]. Weight loss improves these parameters and enhances male fertility.

GLP-1 receptors are expressed in human testicular tissue, including Leydig cells, Sertoli cells, and germ cells [133]. Preclinical studies have demonstrated that GLP-1 receptor activation influences testicular function, though results have been mixed. Some studies reported that GLP-1 receptor agonists improve spermatogenesis, enhance testosterone production, and protect against oxidative stress-induced testicular damage. Other studies found neutral or negative effects on sperm parameters [134].

Clinical evidence for GLP-1 receptor agonists in male fertility is limited. Observational studies in men with type 2 diabetes and obesity treated with GLP-1 receptor agonists have reported improvements in testosterone levels, erectile function, and sexual satisfaction [134]. These improvements likely reflect both weight loss and improved metabolic health.

For semaglutide specifically, a small study of men with obesity and hypogonadism found that semaglutide treatment was associated with increases in total testosterone, improvements in erectile function scores, and enhanced libido. Another study reported improvements in semen parameters including sperm concentration and motility in men with obesity treated with semaglutide.

However, concerns have been raised about potential adverse effects of GLP-1 receptor agonists on male fertility. Rapid weight loss can transiently impair spermatogenesis, and the appetite suppression associated with these agents may lead to inadequate protein and micronutrient intake, potentially affecting sperm production. Additionally, preclinical studies have reported that high doses of GLP-1 receptor agonists can impair testicular function in some animal models [48].

For tirzepatide, data on male fertility are essentially absent. The dual GIP/GLP-1 receptor agonism may have distinct effects on testicular function compared with selective GLP-1 receptor agonists, but this is entirely speculative.

Men planning conception should be counseled about the potential effects of GLP-1 receptor agonists on fertility. While current evidence suggests that these agents are more likely to improve than impair male fertility through weight loss and metabolic improvement, individual responses may vary. Monitoring of testosterone levels and semen parameters may be appropriate in men with fertility concerns.

### 7.3. Pregnancy Safety and Periconception Considerations

Both semaglutide and tirzepatide are contraindicated during pregnancy based on animal reproductive toxicity studies. In animal studies, GLP-1 receptor agonists caused embryofetal toxicity, including increased rates of fetal malformations, growth restriction, and pregnancy loss, at clinically relevant exposures [48,135].

Regarding pregnancy safety: both semaglutide and tirzepatide are currently contraindicated during pregnancy based on animal teratogenicity data demonstrating embryofetal toxicity at clinically relevant exposures. Human data are limited to pharmacovigilance reports and small observational studies, which are insufficient to characterize reproductive risk. Women of childbearing potential should be counseled to discontinue these agents at least 2 months before planned conception (semaglutide) given the prolonged half-life. Long-term reproductive outcomes, effects on offspring development, and safety during lactation remain unstudied. The manuscript should not be interpreted as endorsing periconception use of these agents.

Human data on pregnancy outcomes with GLP-1 receptor agonist exposure are limited. Pharmacovigilance reports and case series have described pregnancies exposed to GLP-1 receptor agonists, with variable outcomes [136,137]. Some reports described normal pregnancies and healthy infants, while others reported adverse outcomes including miscarriage, congenital anomalies, and preterm birth [138]. However, these observational data are insufficient to establish causality, as women with diabetes and obesity have elevated baseline risks for adverse pregnancy outcomes [137].

A recent systematic review of pregnancy exposures to GLP-1 receptor agonists found no consistent pattern of congenital anomalies, but noted that data were limited and subject to reporting bias [131]. The review concluded that current evidence is insufficient to characterize the reproductive risk of GLP-1 receptor agonists in humans.

Given the long half-life of semaglutide (approximately 1 week) and tirzepatide (approximately 5 days), these agents should be discontinued well in advance of planned conception to allow for drug clearance. Current recommendations suggest discontinuing semaglutide at least 2 months before planned conception [130]. For tirzepatide, similar recommendations are appropriate given its pharmacokinetic profile.

The improved fertility associated with GLP-1 receptor agonist treatment in women with PCOS and obesity raises concerns about unintended pregnancy. Women of reproductive potential should be counseled about this risk and advised to use effective contraception during treatment [131]. Pregnancy testing should be considered if menses are missed or pregnancy is suspected.

If pregnancy occurs during GLP-1 receptor agonist treatment, the medication should be discontinued immediately. The patient should be referred for obstetric care and counseling about potential risks. Close monitoring throughout pregnancy is appropriate, though the specific risks associated with early pregnancy exposure remain uncertain [48].

Regarding lactation, it is unknown whether semaglutide or tirzepatide are excreted in human breast milk. Animal studies have detected GLP-1 receptor agonists in milk, raising concerns about infant exposure [136]. Given the lack of human data, breastfeeding is not recommended during treatment with these agents [139].

The periconception use of GLP-1 receptor agonists presents a clinical dilemma. On one hand, weight loss and metabolic improvement before pregnancy are beneficial and may reduce risks of gestational diabetes, preeclampsia, and other obesity-related pregnancy complications [131]. On the other hand, the potential teratogenic risks of these agents necessitate discontinuation before conception. The optimal approach involves initiating GLP-1 receptor agonist therapy well in advance of planned pregnancy, achieving weight loss and metabolic improvement, discontinuing the medication at least 2 months before conception, and maintaining weight loss through lifestyle interventions during the periconception period and pregnancy [7].

### 7.4. Clinical Implications and Future Directions

GLP-1 receptor agonists represent a promising therapeutic option for women with PCOS and obesity, and may improve fertility outcomes through weight loss and metabolic improvement. However, the reproductive safety concerns necessitate careful patient selection, counseling, and monitoring.

Key clinical recommendations include:

1. Women of reproductive potential should be counseled about the potential for improved fertility and the need for effective contraception during treatment.
2. Women planning pregnancy should discontinue GLP-1 receptor agonists at least 2 months before conception.
3. If pregnancy occurs during treatment, the medication should be discontinued immediately and the patient referred for obstetric care.
4. Men with fertility concerns should be counseled about potential effects on fertility, though current evidence suggests benefits are more likely than harms.
5. Breastfeeding is not recommended during treatment with GLP-1 receptor agonists given the lack of safety data.

Future research priorities include:

1. Prospective studies of pregnancy outcomes following GLP-1 receptor agonist exposure to better characterize reproductive risks.
2. Studies of optimal timing of medication discontinuation before conception to balance metabolic benefits with safety concerns.
3. Investigation of direct effects of GLP-1 receptor agonists on ovarian and testicular function, independent of weight loss.
4. Comparative studies of semaglutide and tirzepatide in reproductive populations.
5. Long-term follow-up studies of offspring exposed to GLP-1 receptor agonists during early pregnancy or the periconception period.

## 8. Safety Considerations in Unconventional Applications

The safety profile of semaglutide and tirzepatide has been extensively characterized in clinical trials for their approved indications of type 2 diabetes and obesity [2,8]. However, the use of these agents for unconventional applications raises specific safety considerations that warrant careful attention.

### 8.1. Gastrointestinal Adverse Effects

Gastrointestinal adverse effects are the most common side effects of GLP-1 receptor agonists, occurring in 40–70% of patients [2]. These include nausea, vomiting, diarrhea, constipation, abdominal pain, and dyspepsia [6]. The mechanisms involve delayed gastric emptying, modulation of gastrointestinal motility, and central effects on nausea and vomiting centers [2].

For unconventional applications, gastrointestinal adverse effects may have specific implications. In patients with cancer, particularly those receiving chemotherapy, the addition of GLP-1 receptor agonist-induced nausea may be poorly tolerated and could exacerbate cancer-related cachexia [2]. In patients with eating disorders or addiction, gastrointestinal symptoms might be misinterpreted as withdrawal symptoms or could complicate nutritional rehabilitation [2].

Strategies to mitigate gastrointestinal adverse effects include gradual dose escalation, taking the medication with food, avoiding large or high-fat meals, and use of antiemetic medications when necessary [2]. Most gastrointestinal symptoms improve over time as tolerance develops [38].

### 8.2. Pancreatitis and Pancreatic Safety

Concerns about pancreatitis risk with GLP-1 receptor agonists emerged from early case reports and preclinical studies [40]. However, large-scale clinical trials and meta-analyses have not demonstrated increased pancreatitis risk with these agents [41,117]. The SUSTAIN and PIONEER trials of semaglutide reported pancreatitis rates similar to placebo [15,42]. The SURPASS trials of tirzepatide also found no increased pancreatitis risk [2].

Nevertheless, GLP-1 receptor agonists should be used with caution in patients with a history of pancreatitis, and patients should be counseled to seek medical attention if they develop severe abdominal pain [17]. For patients with pancreatic cancer, the theoretical concerns about GLP-1 receptor effects on pancreatic tissue warrant careful consideration, though current evidence does not support increased cancer risk [48].

### 8.3. Thyroid Safety

As discussed in the oncology section, GLP-1 receptor agonists are contraindicated in patients with personal or family history of medullary thyroid carcinoma or multiple endocrine neoplasia type 2, based on rodent findings of C-cell tumors [48]. However, human data have not demonstrated increased thyroid cancer risk [50].

Patients should be counseled about symptoms of thyroid tumors (neck mass, dysphagia, dyspnea, persistent hoarseness) and instructed to report these symptoms promptly [48]. Routine thyroid monitoring is not recommended in the absence of symptoms or risk factors [14].

### 8.4. Cardiovascular Effects

GLP-1 receptor agonists have demonstrated cardiovascular benefits in outcome trials, including reductions in major adverse cardiovascular events [15,20]. These benefits extend to patients without established cardiovascular disease [14]. For unconventional applications, the cardiovascular effects are generally favorable, though specific populations require consideration.

In patients with cancer receiving cardiotoxic chemotherapy, the cardiovascular protective effects of GLP-1 receptor agonists might be beneficial [82]. In patients with substance use disorders, particularly those using stimulants, the cardiovascular effects of GLP-1 receptor agonists should be considered in the context of drug-induced cardiovascular stress [2].

### 8.5. Hypoglycemia

GLP-1 receptor agonists have low intrinsic hypoglycemia risk due to their glucose-dependent mechanism of action [2]. However, hypoglycemia can occur when these agents are combined with insulin or sulfonylureas [2]. For patients without diabetes using GLP-1 receptor agonists for unconventional indications, hypoglycemia risk is minimal [14].

### 8.6. Renal Effects

GLP-1 receptor agonists have demonstrated renal protective effects in clinical trials, including reductions in albuminuria and slowing of estimated glomerular filtration rate (eGFR) decline [2,15]. These agents can be used in patients with chronic kidney disease, though dose adjustments may be necessary for some agents [48]. Semaglutide and tirzepatide do not require dose adjustment for renal impairment [2,48].

Acute kidney injury has been reported in association with GLP-1 receptor agonists, typically in the context of severe gastrointestinal adverse effects leading to dehydration [2]. Patients should be counseled about the importance of maintaining adequate hydration, particularly during the initial weeks of treatment [79].

### 8.7. Hepatobiliary Effects

GLP-1 receptor agonists have beneficial effects on liver health, including improvements in hepatic steatosis, inflammation, and fibrosis in patients with NAFLD/NASH [74,79]. However, these agents are associated with increased risk of cholelithiasis and cholecystitis, likely related to rapid weight loss [74].

Patients should be counseled about symptoms of gallbladder disease (right upper quadrant pain, nausea, vomiting) and instructed to seek medical attention if these occur [74]. For patients with a history of gallbladder disease, the risks and benefits of GLP-1 receptor agonist therapy should be carefully considered [15].

### 8.8. Retinopathy

In the SUSTAIN-6 trial, semaglutide was associated with increased risk of diabetic retinopathy complications compared with placebo [15]. This finding was attributed to rapid improvement in glycemic control in patients with pre-existing retinopathy [7]. Subsequent trials with more gradual glycemic improvement did not demonstrate increased retinopathy risk [15].

For patients with diabetes and pre-existing retinopathy, gradual dose escalation and close ophthalmologic monitoring are recommended [15]. For patients without diabetes using GLP-1 receptor agonists for unconventional indications, retinopathy risk is not a concern [19].

### 8.9. Psychiatric and Neurological Effects

Concerns about psychiatric effects of GLP-1 receptor agonists, particularly suicidal ideation, emerged from pharmacovigilance reports [19]. However, large-scale analyses of clinical trial data have not demonstrated increased risk of suicidal ideation or behavior with these agents [19,102]. Some studies have reported improvements in mood and quality of life with GLP-1 receptor agonist treatment [19].

For patients with pre-existing psychiatric conditions, particularly depression or suicidal ideation, close monitoring is appropriate when initiating GLP-1 receptor agonist therapy [19]. The potential benefits of these agents for addiction and eating disorders should be weighed against theoretical psychiatric risks [2].

### 8.10. Injection Site Reactions

Injection site reactions, including erythema, pruritus, and induration, occur in approximately 1–5% of patients treated with GLP-1 receptor agonists [2]. These reactions are typically mild and resolve spontaneously [2]. Strategies to minimize injection site reactions include rotating injection sites, allowing the medication to reach room temperature before injection, and proper injection technique [2].

A comprehensive safety evaluation of semaglutide and tirzepatide in unconventional applications must address several clinically important concerns beyond gastrointestinal adverse effects. First, substantial weight loss induced by these agents is associated with significant lean mass reduction, raising concerns about sarcopenia, particularly in older adults, patients with cancer-related cachexia, and individuals with pre-existing muscle mass deficits. Concurrent resistance exercise and adequate protein intake are advisable to mitigate this risk, though evidence-based protocols in these specific populations are lacking. Second, marked appetite suppression may predispose to nutritional deficiencies including inadequate intake of protein, micronutrients, and essential fatty acids, with potential implications for bone health, immune function, and wound healing. Third, gallbladder disease, including cholelithiasis and cholecystitis, occurs with increased frequency during rapid weight loss facilitated by these agents and should be monitored clinically. Fourth, delayed gastric emptying represents a clinically significant concern in perioperative settings, with current anesthesiology guidelines recommending discontinuation of GLP-1 receptor agonists prior to elective procedures to reduce aspiration risk. Fifth, in oncology patients, the appetite-suppressing and weight-reducing effects of these agents may exacerbate cancer-related cachexia, a condition associated with poor prognosis and impaired treatment tolerance; their use in this population requires individualized risk-benefit assessment. Sixth, in psychiatric patients, adherence to weekly injectable therapy may be challenging, and the neuropsychiatric effects of these agents—including reported associations with suicidal ideation in pharmacovigilance databases, though causality remains unestablished—warrant monitoring. Seventh, the socioeconomic dimension of access to these high-cost agents introduces a confounding variable in observational studies: patients able to access semaglutide or tirzepatide may systematically differ from untreated comparators in ways that independently predict better health outcomes, potentially inflating apparent treatment benefits.

### 8.11. Special Population Considerations

Several special populations require specific safety considerations when using GLP-1 receptor agonists for unconventional applications.

Elderly patients: Older adults may be more susceptible to adverse effects including gastrointestinal symptoms, dehydration, and weight loss-associated complications [2]. Sarcopenia risk is particularly concerning in this population [2]. Gradual dose escalation, close monitoring, and emphasis on resistance exercise and adequate protein intake are important [2].

Patients with cancer: The use of GLP-1 receptor agonists in patients with cancer requires careful consideration of potential benefits (metabolic improvement, potential antitumor effects) versus risks (exacerbation of cachexia, interaction with chemotherapy, gastrointestinal symptoms) [2]. Individualized risk-benefit assessment is essential [104].

Patients with psychiatric disorders: While GLP-1 receptor agonists show promise for addiction and eating disorders, patients with severe mental illness may face adherence challenges and require close monitoring for psychiatric adverse effects [136].

Pregnant and lactating women: As discussed in the reproductive medicine section, GLP-1 receptor agonists are contraindicated in pregnancy and not recommended during lactation [48].

Pediatric patients: Semaglutide is approved for adolescents aged 12 and older with obesity, but data on unconventional applications in pediatric populations are lacking [48]. Tirzepatide is not currently approved for pediatric use [2].

### 8.12. Drug Interactions

GLP-1 receptor agonists have relatively few direct drug interactions, but their effects on gastric emptying can influence the absorption of oral medications [2]. Medications requiring rapid absorption or narrow therapeutic windows may be affected [2]. Patients should be counseled to take oral medications at least 1 h before or 4 h after GLP-1 receptor agonist injection [2].

For patients with diabetes, dose adjustments of insulin or sulfonylureas may be necessary to prevent hypoglycemia when initiating GLP-1 receptor agonist therapy [2]. For patients taking warfarin, increased INR monitoring may be appropriate due to potential effects on vitamin K absorption [140].

### 8.13. Long-Term Safety

The long-term safety of semaglutide and tirzepatide beyond 2–3 years is not well established, as most clinical trials have been of shorter duration [140]. Ongoing post-marketing surveillance and long-term extension studies are important for identifying rare or delayed adverse effects [140].

For unconventional applications, where treatment duration may extend for many years, long-term safety monitoring is particularly important. Areas of uncertainty include long-term effects on bone health, potential for tachyphylaxis or tolerance, and effects of prolonged appetite suppression on nutritional status [140].

## 9. Conclusions and Future Directions

Semaglutide and tirzepatide have emerged as transformative therapies for type 2 diabetes and obesity, and accumulating evidence suggests potential applications across diverse therapeutic domains extending far beyond their original indications. This review has synthesized current evidence for unconventional uses of these agents in oncology, psychiatry and addiction medicine, orthopedics, aesthetic medicine, and reproductive medicine.

Several overarching themes emerge from this comprehensive evaluation. First, the pleiotropic effects of GLP-1 and GIP receptor agonists reflect the widespread tissue distribution of these receptors and the diverse signaling pathways they regulate. The mechanistic rationale for unconventional applications is generally strong, supported by receptor expression studies, in vitro experiments, and animal models demonstrating relevant biological effects.

Second, the strength of clinical evidence varies dramatically across different applications. Some domains, such as knee osteoarthritis, are supported by randomized controlled trials demonstrating clinically meaningful benefits. Other domains, such as oncology and addiction medicine, rest primarily on preclinical studies and observational data that, while promising, are subject to substantial limitations including confounding, selection bias, and uncertain translatability from animal models to human disease.

Third, a critical challenge across all domains is distinguishing direct pharmacological effects of GLP-1 receptor agonists from consequences of weight loss and metabolic improvement. Many of the observed benefits in observational studies may reflect the well-established health benefits of weight reduction rather than unique drug effects. Mechanistic studies in non-obese populations and comparative studies with other weight loss interventions are needed to address this question.

Fourth, the distinction between semaglutide and tirzepatide, and between these newer agents and earlier GLP-1 receptor agonists, requires greater attention. Most mechanistic and clinical data derive from studies of liraglutide or exenatide, and extrapolation to semaglutide and particularly to tirzepatide may not be valid. The dual GIP/GLP-1 receptor agonism of tirzepatide may confer distinct effects compared with selective GLP-1 receptor agonists, but this remains largely unexplored. Head-to-head comparative studies are needed to inform agent selection for specific applications.

Fifth, safety considerations specific to unconventional applications require careful attention. While the overall safety profile of these agents is favorable, certain populations (elderly, cancer patients, psychiatric patients) and certain applications (periconception use, use in cachexia) raise specific concerns that necessitate individualized risk-benefit assessment and close monitoring.

Looking forward, several research priorities emerge:

1. Randomized controlled trials for specific unconventional indications, particularly in addiction medicine, oncology, and dermatology, are urgently needed to establish efficacy and safety.
2. Mechanistic studies to elucidate whether observed effects are mediated through direct receptor activation in target tissues, indirect metabolic effects, or both, will inform optimal use and identify patients most likely to benefit.
3. Biomarker studies to identify predictors of treatment response across different applications will enable precision medicine approaches.
4. Comparative effectiveness studies of semaglutide versus tirzepatide, and of GLP-1 receptor agonists versus other interventions, will inform clinical decision-making and resource allocation.
5. Long-term safety studies extending beyond 2–3 years are needed to characterize the safety profile of prolonged treatment for unconventional indications.
6. Health economic analyses to evaluate the cost-effectiveness of GLP-1 receptor agonists for unconventional applications will inform policy decisions and reimbursement.
7. Implementation science studies to understand barriers and facilitators to the appropriate use of these agents for unconventional indications will support the translation of evidence into practice.

The expanding therapeutic potential of semaglutide and tirzepatide represents an exciting frontier in medicine, with implications for millions of patients suffering from conditions currently lacking effective treatments. However, enthusiasm must be tempered by recognition of the limitations of current evidence and the need for rigorous investigation before these agents can be recommended for unconventional applications outside of clinical trials. The coming years will be critical for translating promising preclinical and observational findings into evidence-based clinical practice, ultimately determining whether the unconventional applications of semaglutide and tirzepatide fulfill their therapeutic promise.

## Figures and Tables

**Table 1:** Summary of Evidence Levels by Therapeutic Domain.

Therapeutic Domain	Best Available Evidence	Evidence Level	Key Agents Studied	Strength of Recommendation
Oncology (colorectal cancer)	Observational cohort studies	Level 3	Semaglutide, liraglutide	Exploratory—insufficient for clinical use
Oncology (pancreatic cancer)	Preclinical + conflicting observational	Level 4	GLP-1 RAs (general)	Speculative—conflicting data
Oncology (thyroid cancer)	Pharmacovigilance + preclinical	Level 4	Semaglutide	Speculative
Oncology (breast cancer)	Preclinical + small observational	Level 4	GLP-1 RAs (general)	Speculative
Alcohol Use Disorder	Small RCTs + retrospective cohorts	Level 2–3	Semaglutide, exenatide	Investigational
Substance Use Disorder	Mostly preclinical + pilot studies	Level 4	GLP-1 RAs (general)	Investigational—insufficient for clinical use
Binge-Eating Disorder	Small RCTs + observational	Level 2–3	Semaglutide	Investigational
Knee Osteoarthritis	RCT (STEP-9)	Level 1–2	Semaglutide	Promising—ongoing trials needed
Bone Health	Mixed preclinical + observational	Level 3–4	Semaglutide, tirzepatide	Uncertain
Aesthetic Medicine (facial volume loss)	Case series + expert opinion	Level 5	Semaglutide	Descriptive—no controlled evidence
Inflammatory Dermatoses	Small uncontrolled studies	Level 4–5	GLP-1 RAs (general)	Speculative
PCOS/Female Fertility	Observational + small RCTs	Level 2–3	Semaglutide	Indirect benefit via weight loss
Male Fertility	Preclinical + small observational	Level 4	Semaglutide	Speculative
Pregnancy Safety	Pharmacovigilance + animal data	Level 4–5	Semaglutide	Insufficient—contraindicated in pregnancy

## Data Availability

No new data were created or analyzed in this study. Data sharing is not applicable to this article.

## References

[1] Drucker D.J. (2018). Mechanisms of Action and Therapeutic Application of Glucagon-like Peptide-1. Cell Metab..

[2] Nauck M.A., Meier J.J. (2018). Incretin hormones: Their role in health and disease. Diabetes Obes. Metab..

[3] Lau J., Bloch P., Schäffer L., Pettersson I., Spetzler J., Kofoed J., Madsen K., Knudsen L.B., McGuire J., Steensgaard D.B. (2015). Discovery of the Once-Weekly Glucagon-Like Peptide-1 (GLP-1) Analogue Semaglutide. J. Med. Chem..

[4] Frias J.P., Nauck M.A., Van J., Kutner M.E., Cui X., Benson C., Urva S., Gimeno R.E., Milicevic Z., Robins D. (2018). Efficacy and safety of LY3298176, a novel dual GIP and GLP-1 receptor agonist, in patients with type 2 diabetes: A randomised, placebo-controlled and active comparator-controlled phase 2 trial. Lancet.

[5] Kapitza C., Nosek L., Jensen L., Hartvig H., Jensen C.B., Flint A. (2015). Semaglutide, a once-weekly human GLP-1 analog, does not reduce the bioavailability of the combined oral contraceptive, ethinylestradiol/levonorgestrel. J. Clin. Pharmacol..

[6] Urva S., Coskun T., Loghin C., Cui X., Beebe E., O’Farrell L., Briere D.A., Benson C.T., Nauck M.A., Haupt A. (2022). The novel dual glucose-dependent insulinotropic polypeptide and glucagon-like peptide-1 (GIP/GLP-1) receptor agonist tirzepatide transiently delays gastric emptying similarly across subjects with type 2 diabetes, obesity, and healthy body weight. Diabetes Obes. Metab..

[7] Wilding J.P.H., Batterham R.L., Calanna S., Davies M., Van Gaal L.F., Lingvay I., McGowan B.M., Rosenstock J., Tran M.T.D., Wadden T.A. (2021). Once-Weekly Semaglutide in Adults with Overweight or Obesity. N. Engl. J. Med..

[8] Jastreboff A.M., Aronne L.J., Ahmad N.N., Wharton S., Connery L., Alves B., Kiyosue A., Zhang S., Liu B., Bunck M.C. (2022). Tirzepatide Once Weekly for the Treatment of Obesity. N. Engl. J. Med..

[9] Pyke C., Heller R.S., Kirk R.K., Ørskov C., Reedtz-Runge S., Kaastrup P., Hvelplund A., Bardram L., Calatayud D., Knudsen L.B. (2014). GLP-1 receptor localization in monkey and human tissue: Novel distribution revealed with extensively validated monoclonal antibody. Endocrinology.

[10] Yip R.G., Wolfe M.M. (2000). GIP biology and fat metabolism. Life Sci..

[11] Holst J.J. (2007). The physiology of glucagon-like peptide 1. Physiol. Rev..

[12] Baggio L.L., Drucker D.J. (2007). Biology of incretins: GLP-1 and GIP. Gastroenterology.

[13] Koperdowska J.M., Broen J.A. (2026). Pleiotropic Effects of GLP-1 Receptor Agonists—A Narrative Review of Extra-Metabolic Outcomes in Adults. Med. Res. J..

[14] Marso S.P., Daniels G.H., Brown-Frandsen K., Kristensen P., Mann J.F., Nauck M.A., Nissen S.E., Pocock S., Poulter N.R., Ravn L.S. (2016). Liraglutide and Cardiovascular Outcomes in Type 2 Diabetes. N. Engl. J. Med..

[15] Marso S.P., Bain S.C., Consoli A., Eliaschewitz F.G., Jódar E., Leiter L.A., Lingvay I., Rosenstock J., Seufert J., Warren M.L. (2016). Semaglutide and Cardiovascular Outcomes in Patients with Type 2 Diabetes. N. Engl. J. Med..

[16] Cork S.C., Richards J.E., Holt M.K., Gribble F.M., Reimann F., Trapp S. (2015). Distribution and characterisation of Glucagon-like peptide-1 receptor expressing cells in the mouse brain. Mol. Metab..

[17] Koehler J.A., Baggio L.L., Lamont B.J., Ali S., Drucker D.J. (2009). Glucagon-like peptide-1 receptor activation modulates pancreatitis-associated gene expression but does not modify the susceptibility to experimental pancreatitis in mice. Diabetes.

[18] Athauda D., Maclagan K., Skene S.S., Bajwa-Joseph M., Letchford D., Chowdhury K., Hibbert S., Budnik N., Zampedri L., Dickson J. (2017). Exenatide once weekly versus placebo in Parkinson’s disease: A randomised, double-blind, placebo-controlled trial. Lancet.

[19] Wang W., Volkow N.D., Berger N.A., Davis P.B., Kaelber D.C., Xu R. (2024). Association of semaglutide with risk of suicidal ideation in a real-world cohort. Nat. Med..

[20] Kosiborod M.N., Abildstrøm S.Z., Borlaug B.A., Butler J., Rasmussen S., Davies M., Hovingh G.K., Kitzman D.W., Lindegaard M.L., Møller D.V. (2023). Semaglutide in Patients with Heart Failure with Preserved Ejection Fraction and Obesity. N. Engl. J. Med..

[21] Körner M., Stöckli M., Waser B., Reubi J.C. (2007). GLP-1 receptor expression in human tumors and human normal tissues: Potential for in vivo targeting. J. Nucl. Med..

[22] Ligumsky H., Wolf I., Israeli S., Haimsohn M., Ferber S., Karasik A., Kaufman B., Rubinek T. (2012). The peptide-hormone glucagon-like peptide-1 activates cAMP and inhibits growth of breast cancer cells. Breast Cancer Res. Treat..

[23] Koehler J.A., Kain T., Drucker D.J. (2011). Glucagon-like peptide-1 receptor activation inhibits growth and augments apoptosis in murine CT26 colon cancer cells. Endocrinology.

[24] Zhao H., Wang L., Wei R., Xiu D., Tao M., Ke J., Liu Y., Yang J., Hong T. (2014). Activation of glucagon-like peptide-1 receptor inhibits growth and promotes apoptosis of human pancreatic cancer cells in a cAMP-dependent manner. Am. J. Physiol. Endocrinol. Metab..

[25] Iwaya C., Nomiyama T., Komatsu S., Kawanami T., Tsutsumi Y., Hamaguchi Y., Horikawa T., Yoshinaga Y., Yamashita S., Tanaka T. (2017). Exendin-4, a Glucagonlike Peptide-1 Receptor Agonist, Attenuates Breast Cancer Growth by Inhibiting NF-κB Activation. Endocrinology.

[26] Hanahan D., Weinberg R.A. (2011). Hallmarks of cancer: The next generation. Cell.

[27] Sato A., Kawano H., Notsu T., Ohta M., Nakakuki M., Mizuguchi K., Itoh M., Suganami T., Ogawa Y. (2010). Antiobesity effect of eicosapentaenoic acid in high-fat/high-sucrose diet-induced obesity: Importance of hepatic lipogenesis. Diabetes.

[28] Sharma D., Verma S., Vaidya S., Kalia K., Tiwari V. (2018). Recent updates on GLP-1 agonists: Current advancements & challenges. Biomed. Pharmacother..

[29] Hogan A.E., Gaoatswe G., Lynch L., Corrigan M.A., Woods C., O’Connell J., O’Shea D. (2014). Glucagon-like peptide 1 analogue therapy directly modulates innate immune-mediated inflammation in individuals with type 2 diabetes mellitus. Diabetologia.

[30] Rakoff-Nahoum S., Medzhitov R. (2009). Toll-like receptors and cancer. Nat. Rev. Cancer.

[31] Krasner N.M., Ido Y., Ruderman N.B., Cacicedo J.M. (2014). Glucagon-like peptide-1 (GLP-1) analog liraglutide inhibits endothelial cell inflammation through a calcium and AMPK dependent mechanism. PLoS ONE.

[32] Grivennikov S.I., Greten F.R., Karin M. (2010). Immunity, inflammation, and cancer. Cell.

[33] Erdogdu Ö., Nathanson D., Sjöholm Å., Nyström T., Zhang Q. (2010). Exendin-4 stimulates proliferation of human coronary artery endothelial cells through eNOS-, PKA- and PI3K/Akt-dependent pathways and requires GLP-1 receptor. Mol. Cell. Endocrinol..

[34] Carmeliet P., Jain R.K. (2000). Angiogenesis in cancer and other diseases. Nature.

[35] Tsutsumi Y., Nomiyama T., Kawanami T., Hamaguchi Y., Terawaki Y., Tanaka T., Murase K., Motonaga R., Tanabe M., Yanase T. (2015). Combined Treatment with Exendin-4 and Metformin Attenuates Prostate Cancer Growth. PLoS ONE.

[36] Elmore S. (2007). Apoptosis: A review of programmed cell death. Toxicol. Pathol..

[37] Siegel R.L., Miller K.D., Wagle N.S., Jemal A. (2023). Cancer statistics, 2023. CA Cancer J. Clin..

[38] Elashoff M., Matveyenko A.V., Gier B., Elashoff R., Butler P.C. (2011). Pancreatitis, pancreatic, and thyroid cancer with glucagon-like peptide-1-based therapies. Gastroenterology.

[39] Gier B., Matveyenko A.V., Kirakossian D., Dawson D., Dry S.M., Butler P.C. (2012). Chronic GLP-1 receptor activation by exendin-4 induces expansion of pancreatic duct glands in rats and accelerates formation of dysplastic lesions and chronic pancreatitis in the Kras(G12D) mouse model. Diabetes.

[40] Giorda C.B., Nada E., Tartaglino B., Marafetti L., Gnavi R. (2014). A systematic review of acute pancreatitis as an adverse event of type 2 diabetes drugs: From hard facts to a balanced position. Diabetes Obes. Metab..

[41] Monami M., Nreu B., Scatena A., Cresci B., Andreozzi F., Sesti G., Mannucci E. (2017). Safety issues with glucagon-like peptide-1 receptor agonists (pancreatitis, pancreatic cancer and cholelithiasis): Data from randomized controlled trials. Diabetes Obes. Metab..

[42] Husain M., Birkenfeld A.L., Donsmark M., Dungan K., Eliaschewitz F.G., Franco D.R., Jeppesen O.K., Lingvay I., Mosenzon O., Pedersen S.D. (2019). Oral Semaglutide and Cardiovascular Outcomes in Patients with Type 2 Diabetes. N. Engl. J. Med..

[43] Nomiyama T., Kawanami T., Irie S., Hamaguchi Y., Terawaki Y., Murase K., Tsutsumi Y., Nagaishi R., Tanabe M., Morinaga H. (2014). Exendin-4, a GLP-1 receptor agonist, attenuates prostate cancer growth. Diabetes.

[44] Apte M.V., Wilson J.S., Lugea A., Pandol S.J. (2013). A starring role for stellate cells in the pancreatic cancer microenvironment. Gastroenterology.

[45] Jain R.K. (2001). Normalizing tumor vasculature with anti-angiogenic therapy: A new paradigm for combination therapy. Nat. Med..

[46] Kawaguchi T., Nakano D., Koga H., Torimura T. (2019). Effects of a DPP4 inhibitor on progression of NASH-related HCC and the p62/ Keap1/Nrf2-pentose phosphate pathway in a mouse model. Liver Cancer.

[47] Bjerre Knudsen L., Madsen L.W., Andersen S., Almholt K., de Boer A.S., Drucker D.J., Gotfredsen C., Egerod F.L., Hegelund A.C., Jacobsen H. (2010). Glucagon-like Peptide-1 receptor agonists activate rodent thyroid C-cells causing calcitonin release and C-cell proliferation. Endocrinology.

[48] U.S. Food and Drug Administration Ozempic (Semaglutide) Injection, for Subcutaneous Use: Prescribing Information. https://www.accessdata.fda.gov/drugsatfda_docs/label/2017/209637lbl.pdf.

[49] Gier B., Butler P.C., Lai C.K., Kirakossian D., DeNicola M.M., Yeh M.W. (2012). Glucagon like peptide-1 receptor expression in the human thyroid gland. J. Clin. Endocrinol. Metab..

[50] Bezin J., Gouverneur A., Pénichon M., Mathieu C., Garrel R., Hillaire-Buys D., Pariente A., Faillie J.L. (2023). GLP-1 Receptor Agonists and the Risk of Thyroid Cancer. Diabetes Care.

[51] Faillie J.L., Yu O.H., Yin H., Hillaire-Buys D., Barkun A., Azoulay L. (2016). Association of Bile Duct and Gallbladder Diseases with the Use of Incretin-Based Drugs in Patients with Type 2 Diabetes Mellitus. JAMA Intern. Med..

[52] Silverii G.A., Monami M., Mannucci E. (2024). Glucagon-like peptide-1 receptor agonists and risk of thyroid cancer: A systematic review and meta-analysis of randomized controlled trials. Diabetes Obes. Metab..

[53] Waser B., Beetschen K., Pellegata N.S., Reubi J.C. (2011). Incretin receptors in non-neoplastic and neoplastic thyroid C cells in rodents and humans: Relevance to calcitonin and medullary thyroid carcinoma. Neuroendocrinology.

[54] Zhang X., Zhang L., Wang B., Zhang X., Gu L., Guo K., Zhang X., Zhou Z. (2023). GLP-1 receptor agonist liraglutide inhibits the proliferation and migration of thyroid cancer cells. Cell. Mol. Biol..

[55] Kim S.J., Nian C., McIntosh C.H. (2007). Activation of lipoprotein lipase by glucose-dependent insulinotropic polypeptide in adipocytes. A role for a protein kinase B, LKB1, and AMP-activated protein kinase cascade. J. Biol. Chem..

[56] European Medicines Agency Ozempic (Semaglutide): EPAR—Product Information. https://www.ema.europa.eu/en/medicines/human/EPAR/ozempic.

[57] Sung H., Ferlay J., Siegel R.L., Laversanne M., Soerjomataram I., Jemal A., Bray F. (2021). Global Cancer Statistics 2020: GLOBOCAN Estimates of Incidence and Mortality Worldwide for 36 Cancers in 185 Countries. CA Cancer J. Clin..

[58] Neuhouser M.L., Aragaki A.K., Prentice R.L., Manson J.E., Chlebowski R., Carty C.L., Ochs-Balcom H.M., Thomson C.A., Caan B.J., Tinker L.F. (2015). Overweight, Obesity, and Postmenopausal Invasive Breast Cancer Risk: A Secondary Analysis of the Women’s Health Initiative Randomized Clinical Trials. JAMA Oncol..

[59] Rahn S., Zimmermann V., Viol F., Knaack H., Stemmer K., Peters L., Lenk L., Ungefroren H., Saur D., Schäfer H. (2018). Diabetes as risk factor for pancreatic cancer: Hyperglycemia promotes epithelial-mesenchymal-transition and stem cell properties in pancreatic ductal epithelial cells. Cancer Lett..

[60] Zhao X., Wang M., Wen Z., Lu Z., Cui L., Fu C., Xue H., Liu Y., Zhang Y. (2021). GLP-1 receptor agonists: Beyond their pancreatic effects. Front. Endocrinol..

[61] Malin S.K., Kashyap S.R. (2014). Effects of metformin on weight loss: Potential mechanisms. Curr. Opin. Endocrinol. Diabetes Obes..

[62] Renehan A.G., Zwahlen M., Egger M. (2015). Adiposity and cancer risk: New mechanistic insights from epidemiology. Nat. Rev. Cancer.

[63] Ramos-Nino M.E., MacLean C.D., Littenberg B. (2007). Association between cancer prevalence and use of thiazolidinediones: Results from the Vermont Diabetes Information System. BMC Med..

[64] Tseng C.H. (2018). Metformin and risk of hepatocellular carcinoma in patients with type 2 diabetes. Liver Int..

[65] Scully T., Ettela A., LeRoith D., Gallagher E.J. (2021). Obesity, Type 2 Diabetes, and Cancer Risk. Front. Oncol..

[66] Tseng C.H., Lee K.Y., Tseng F.H. (2015). An updated review on cancer risk associated with incretin mimetics and enhancers. J. Environ. Sci. Health C Environ. Carcinog. Ecotoxicol. Rev..

[67] Arnold M., Sierra M.S., Laversanne M., Soerjomataram I., Jemal A., Bray F. (2017). Global patterns and trends in colorectal cancer incidence and mortality. Gut.

[68] Bardou M., Barkun A.N., Martel M. (2013). Obesity and colorectal cancer. Gut.

[69] Yamada C., Yamada Y., Tsukiyama K., Yamada K., Udagawa N., Takahashi N., Tanaka K., Drucker D.J., Seino Y., Inagaki N. (2008). The murine glucagon-like peptide-1 receptor is essential for control of bone resorption. Endocrinology.

[70] Anbazhagan A.N., Thaqi M., Priyamvada S., Jayawardena D., Kumar A., Gujral T., Chatterjee I., Mugarza E., Saksena S., Onyuksel H. (2017). GLP-1 nanomedicine alleviates gut inflammation. Nanomedicine.

[71] Lebrun L.J., Lenaerts K., Kiers D., Pais de Barros J.P., Le Guern N., Plesnik J., Thomas C., Bourgeois T., Dejong C.H.C., Kox M. (2017). Enteroendocrine L cells sense LPS after gut barrier injury to enhance GLP-1 secretion. Cell Rep..

[72] Hsieh J., Longuet C., Maida A., Bahrami J., Xu E., Baker C.L., Brubaker P.L., Drucker D.J., Adeli K. (2009). Glucagon-like peptide-2 increases intestinal lipid absorption and chylomicron production via CD36. Gastroenterology.

[73] Tseng C.H. (2017). Metformin use is associated with a lower risk of colorectal cancer in Taiwanese patients with type 2 diabetes: A retrospective cohort analysis. J. Gastroenterol. Hepatol..

[74] He L., Wang J., Ping F., Yang N., Huang J., Li Y., Xu L., Li W., Zhang H. (2022). Association of glucagon-like peptide-1 receptor agonist use with risk of gallbladder and biliary diseases: A systematic review and meta-analysis of randomized clinical trials. JAMA Intern. Med..

[75] Giovannucci E., Harlan D.M., Archer M.C., Bergenstal R.M., Gapstur S.M., Habel L.A., Pollak M., Regensteiner J.G., Yee D. (2010). Diabetes and cancer: A consensus report. Diabetes Care.

[76] Tseng C.H. (2012). Diabetes, metformin use, and colon cancer: A population-based cohort study in Taiwan. Eur. J. Endocrinol..

[77] Younossi Z.M., Koenig A.B., Abdelatif D., Fazel Y., Henry L., Wymer M. (2016). Global epidemiology of nonalcoholic fatty liver disease-Meta-analytic assessment of prevalence, incidence, and outcomes. Hepatology.

[78] Anstee Q.M., Reeves H.L., Kotsiliti E., Govaere O., Heikenwalder M. (2019). From NASH to HCC: Current concepts and future challenges. Nat. Rev. Gastroenterol. Hepatol..

[79] Newsome P.N., Buchholtz K., Cusi K., Linder M., Okanoue T., Ratziu V., Sanyal A.J., Sejling A.S., Harrison S.A. (2021). NN9931-4296 Investigators. A Placebo-Controlled Trial of Subcutaneous Semaglutide in Nonalcoholic Steatohepatitis. N. Engl. J. Med..

[80] Merchenthaler I., Lane M., Shughrue P. (1999). Distribution of pre-pro-glucagon and glucagon-like peptide-1 receptor messenger RNAs in the rat central nervous system. J. Comp. Neurol..

[81] Alhadeff A.L., Rupprecht L.E., Hayes M.R. (2012). GLP-1 neurons in the nucleus of the solitary tract project directly to the ventral tegmental area and nucleus accumbens to control for food intake. Endocrinology.

[82] Volkow N.D., Wise R.A., Baler R. (2017). The dopamine motive system: Implications for drug and food addiction. Nat. Rev. Neurosci..

[83] Egecioglu E., Steensland P., Fredriksson I., Feltmann K., Engel J.A., Jerlhag E. (2013). The glucagon-like peptide 1 analogue Exendin-4 attenuates alcohol mediated behaviors in rodents. Psychoneuroendocrinology.

[84] Shirazi R.H., Dickson S.L., Skibicka K.P. (2013). Gut peptide GLP-1 and its analogue, Exendin-4, decrease alcohol intake and reward. PLoS ONE.

[85] Suchankova P., Yan J., Schwandt M.L., Stangl B.L., Caparelli E.C., Momenan R., Jerlhag E., Leggio L. (2015). The glucagon-like peptide-1 receptor as a potential treatment target in alcohol use disorder: Evidence from human genetic association studies and a mouse model of alcohol dependence. Transl. Psychiatry.

[86] Kastin A.J., Akerstrom V. (2003). Entry of exendin-4 into brain is rapid but may be limited at high doses. Int. J. Obes. Relat. Metab. Disord..

[87] Gabery S., Salinas C.G., Paulsen S.J., Ahnfelt-Rønne J., Alanentalo T., Baquero A.F., Buckley S.T., Farkas E., Fekete C., Frederiksen K.S. (2020). Semaglutide lowers body weight in rodents via distributed neural pathways. JCI Insight.

[88] Grant B.F., Goldstein R.B., Saha T.D., Chou S.P., Jung J., Zhang H., Pickering R.P., Ruan W.J., Smith S.M., Huang B. (2015). Epidemiology of DSM-5 Alcohol Use Disorder: Results from the National Epidemiologic Survey on Alcohol and Related Conditions III. JAMA Psychiatry.

[89] Kranzler H.R., Soyka M. (2018). Diagnosis and Pharmacotherapy of Alcohol Use Disorder: A Review. JAMA.

[90] Sørensen G., Reddy I.A., Weikop P., Graham D.L., Stanwood G.D., Wortwein G., Galli A., Fink-Jensen A. (2015). The glucagon-like peptide 1 (GLP-1) receptor agonist exendin-4 reduces cocaine self-administration in mice. Physiol. Behav..

[91] Zhang Y., Liu C., Zhao Y., Zhang X., Li B., Cui R. (2015). The Effects of Calorie Restriction in Depression and Potential Mechanisms. Curr. Neuropharmacol..

[92] Klausen M.K., Thomsen M., Wortwein G., Fink-Jensen A. (2022). The role of glucagon-like peptide 1 (GLP-1) in addictive disorders. Br. J. Pharmacol..

[93] ClinicalTrials.gov Semaglutide for Alcohol Use Disorder. NCT05891587. NCT05891587.

[94] Hernandez N.S., Schmidt H.D. (2019). Central GLP-1 receptors: Novel molecular targets for cocaine use disorder. Physiol. Behav..

[95] American Psychiatric Association (2013). Diagnostic and Statistical Manual of Mental Disorders.

[96] Gearhardt A.N., Corbin W.R., Brownell K.D. (2009). Preliminary validation of the Yale Food Addiction Scale. Appetite.

[97] van Can J., Sloth B., Jensen C.B., Flint A., Blaak E.E., Saris W.H. (2014). Effects of the once-daily GLP-1 analog liraglutide on gastric emptying, glycemic parameters, appetite and energy metabolism in obese, non-diabetic adults. Int. J. Obes..

[98] Blundell J., Finlayson G., Axelsen M., Flint A., Gibbons C., Kvist T., Hjerpsted J.B. (2017). Effects of once-weekly semaglutide on appetite, energy intake, control of eating, food preference and body weight in subjects with obesity. Diabetes Obes. Metab..

[99] Dickson S.L., Shirazi R.H., Hansson C., Bergquist F., Nissbrandt H., Skibicka K.P. (2012). The glucagon-like peptide 1 (GLP-1) analogue, exendin-4, decreases the rewarding value of food: A new role for mesolimbic GLP-1 receptors. J. Neurosci..

[100] Robert S.A., Rohana A.G., Shah S.A., Chinna K., Wan Mohamud W.N., Kamaruddin N.A. (2015). Improvement in binge eating in non-diabetic obese individuals after 3 months of treatment with liraglutide—A pilot study. Obes. Res. Clin. Pract..

[101] Anderberg R.H., Richard J.E., Hansson C., Nissbrandt H., Bergquist F., Skibicka K.P. (2016). GLP-1 is both anxiogenic and antidepressant; divergent effects of acute and chronic GLP-1 on emotionality. Psychoneuroendocrinology.

[102] Mansur R.B., Ahmed J., Cha D.S., Woldeyohannes H.O., Subramaniapillai M., Lovshin J., Lee J.G., Lee J.H., Brietzke E., Reininghaus E.Z. (2017). Liraglutide promotes improvements in objective measures of cognitive dysfunction in individuals with mood disorders: A pilot, open-label study. J. Affect. Disord..

[103] ClinicalTrials.gov Evaluating Liraglutide in Alzheimer’s Disease (ELAD). NCT01843075. NCT01843075.

[104] Ishøy P.L., Knop F.K., Broberg B.V., Bak N., Andersen U.B., Jørgensen N.R., Holst J.J., Fagerlund B., Rostrup E., Glenthøj B.Y. (2017). Effect of GLP-1 receptor agonist treatment on body weight in obese antipsychotic-treated patients with schizophrenia: A randomized, placebo-controlled trial. Diabetes Obes. Metab..

[105] Hunter D.J., Bierma-Zeinstra S. (2019). Osteoarthritis. Lancet.

[106] Bliddal H., Leeds A.R., Christensen R. (2014). Osteoarthritis, obesity and weight loss: Evidence, hypotheses and horizons—A scoping review. Obes. Rev..

[107] Bliddal H., Bays H., Czernichow S., Udsen F.W., Lund M.T., Skov J., Thomsen M., Verma S., Wharton S. (2024). Once-weekly semaglutide in persons with obesity and knee osteoarthritis. N. Engl. J. Med..

[108] Messier S.P., Gutekunst D.J., Davis C., DeVita P. (2005). Weight loss reduces knee-joint loads in overweight and obese older adults with knee osteoarthritis. Arthritis Rheum..

[109] Berenbaum F., Eymard F., Houard X. (2013). Osteoarthritis, inflammation and obesity. Curr. Opin. Rheumatol..

[110] Pereira M., Jeyabalan J., Jørgensen C.S., Hopkinson M., Al-Jazzar A., Roux C.H., Schieker M., Bab I., Chenu C., Apparailly F. (2018). Common signalling pathways in macrophage and osteoclast multinucleation. J. Cell Sci..

[111] Bollag R.J., Zhong Q., Phillips P., Min L., Zhong L., Cameron R., Mulloy A.L., Rasmussen H., Qin F., Ding K.H. (2000). Osteoblast-derived cells express functional glucose-dependent insulinotropic peptide receptors. Endocrinology.

[112] Nuche-Berenguer B., Moreno P., Esbrit P., Dapía S., Caeiro J.R., Cancelas J., Haro-Mora J.J., Villanueva-Peñacarrillo M.L. (2009). Effect of GLP-1 treatment on bone turnover in normal, type 2 diabetic, and insulin-resistant states. Calcif. Tissue Int..

[113] Su B., Sheng H., Zhang M., Bu L., Yang P., Li L., Li F., Sheng C., Han Y., Qu S. (2015). Risk of bone fractures associated with glucagon-like peptide-1 receptor agonists’ treatment: A meta-analysis of randomized controlled trials. Endocrine.

[114] Zhang Y.S., Zheng Y.D., Yuan Y., Chen S.C., Xie B.C. (2021). Effects of Anti-Diabetic Drugs on Fracture Risk: A Systematic Review and Network Meta-Analysis. Front. Endocrinol..

[115] Iepsen E.W., Lundgren J.R., Hartmann B., Pedersen O., Hansen T., Jørgensen N.R., Jensen J.E.B., Holst J.J., Madsbad S., Torekov S.S. (2015). GLP-1 Receptor Agonist Treatment Increases Bone Formation and Prevents Bone Loss in Weight-Reduced Obese Women. J. Clin. Endocrinol. Metab..

[116] Billington E.O., Grey A., Bolland M.J. (2015). The effect of thiazolidinediones on bone mineral density and bone turnover: Systematic review and meta-analysis. Diabetologia.

[117] Rosenstock J., Wysham C., Frías J.P., Kaneko S., Lee C.J., Fernández Landó L., Mao H., Cui X., Karanikas C.A., Thieu V.T. (2021). Efficacy and safety of a novel dual GIP and GLP-1 receptor agonist tirzepatide in patients with type 2 diabetes (SURPASS-1): A double-blind, randomised, phase 3 trial. Lancet.

[118] Funt D., Pavicic T. (2013). Dermal fillers in aesthetics: An overview of adverse events and treatment approaches. Clin. Cosmet. Investig. Dermatol..

[119] Illouz Y.G., Sterodimas A. (2009). Autologous fat transplantation to the breast: A personal technique with 25 years of experience. Aesthet. Plast. Surg..

[120] Rohrich R.J., Pessa J.E. (2007). The fat compartments of the face: Anatomy and clinical implications for cosmetic surgery. Plast. Reconstr. Surg..

[121] Naldi L., Addis A., Chimenti S., Giannetti A., Picardo M., Tomino C., Maccarone M., Chatenoud L., Bertuccio P., Caggese E. (2008). Impact of body mass index and obesity on clinical response to systemic treatment for psoriasis. Evidence from the Psocare project. Dermatology.

[122] Jensen P., Zachariae C., Christensen R., Geiker N.R., Schaadt B.K., Stender S., Hansen P.R., Astrup A., Skov L. (2013). Effect of Weight Loss on the Severity of Psoriasis: A Randomized Clinical Study. JAMA Dermatol..

[123] Alikhan A., Sayed C., Alavi A., Alhusayen R., Brassard A., Burkhart C., Crowell K., Eisen D.B., Gottlieb A.B., Hamzavi I. (2019). North American clinical management guidelines for hidradenitis suppurativa: A publication from the United States and Canadian Hidradenitis Suppurativa Foundations: Part I: Diagnosis, evaluation, and the use of complementary and procedural management. J. Am. Acad. Dermatol..

[124] Azziz R., Carmina E., Chen Z., Dunaif A., Laven J.S., Legro R.S., Lizneva D., Natterson-Horowtiz B., Teede H.J., Yildiz B.O. (2016). Polycystic ovary syndrome. Nat. Rev. Dis. Prim..

[125] Teede H.J., Misso M.L., Costello M.F., Dokras A., Laven J., Moran L., Piltonen T., Norman R.J., International PCOS Network (2018). Recommendations from the international evidence-based guideline for the assessment and management of polycystic ovary syndrome. Hum. Reprod..

[126] Jensterle M., Janez A. (2026). Incretin-Based Anti-obesity Medications in Polycystic Ovary Syndrome: The Evidence Map. Drugs.

[127] Voros C., Chatzinikolaou F., Papapanagiotou I., Polykalas S., Mavrogianni D., Koulakmanidis A.-M., Athanasiou D., Kanaka V., Bananis K., Athanasiou A. (2026). A Systematic Review on GLP-1 Receptor Agonists in Reproductive Health: Integrating IVF Data, Ovarian Physiology and Molecular Mechanisms. Int. J. Mol. Sci..

[128] Jensterle M., Kravos N.A., Goričar K., Janez A. (2017). Short-term effectiveness of low dose liraglutide in combination with metformin versus high dose liraglutide alone in treatment of obese PCOS: Randomized trial. BMC Endocr. Disord..

[129] Elkind-Hirsch K., Marrioneaux O., Bhushan M., Vernor D., Bhushan R. (2008). Comparison of single and combined treatment with exenatide and metformin on menstrual cyclicity in overweight women with polycystic ovary syndrome. J. Clin. Endocrinol. Metab..

[130] Sills E.S., Gatimu J.M., Goodman L.R., Ahmady A., Walsh A.P.H. (2025). Semaglutide and human reproduction: Caution at the intersection of energy balance, ovarian function, and follicular development. Reprod. Biol. Endocrinol..

[131] Dilbaz B., Ateş Ç. (2026). The effects of glucagon-like peptide-1 receptor agonists on fertility, contraception, and pregnancy: Clinical perspectives. Eur. J. Contracept. Reprod. Health Care.

[132] Sermondade N., Faure C., Fezeu L., Shayeb A.G., Bonde J.P., Jensen T.K., Van Wely M., Cao J., Martini A.C., Eskandar M. (2013). BMI in relation to sperm count: An updated systematic review and collaborative meta-analysis. Hum. Reprod. Update.

[133] Cannarella R., Calogero A.E., Condorelli R.A., Greco E.A., Aversa A., La Vignera S. (2021). Is there a role for glucagon-like peptide-1 receptor agonists in the treatment of male infertility?. Andrology.

[134] Deameh M.G., Ramez M., Rowaiee R., Bani Irshid B.A., Mohamed H., Abdelshafi A., Al-osoufi M.A., Mohamed T., Hegazin S.B., Raheem O. (2026). Effects of glucagon-like peptide-1 receptor agonists on male reproductive hormones, semen parameters, and metabolic outcomes: A systematic review. J. Sex. Med..

[135] Thurmond D.C., Baillargeon J.P., Grogan T.R., Bonner-Weir S. (2026). Semaglutide Induces Oxidative Stress and Differentially Modulates mTOR-Dependent Growth and Invasion in Human Trophoblast Cell Models: Implications for Placental Function. Curr. Issues Mol. Biol..

[136] Zipursky J.S., Grewal E., Kahan M., Mamdani M., Juurlink D.N. (2024). Glucagon-like peptide-1 receptor agonists during pregnancy and lactation. CMAJ.

[137] Alfaiz A.A. (2025). GLP-1 receptor agonists and preconception planning: Bridging the gap between obesity treatment and reproductive safety, a narrative review. Ann. Med. Surg..

[138] Provost M.P., Acharya K.S., Acharya C.R., Yeh J.S., Steward R.G., Eaton J.L., Goldfarb J.M., Muasher S.J. (2016). Pregnancy outcomes decline with increasing body mass index: Analysis of 239,127 fresh autologous in vitro fertilization cycles from the 2008–2010 Society for Assisted Reproductive Technology registry. Fertil. Steril..

[139] Sim K.A., Partridge S.R., Sainsbury A. (2014). Does weight loss in overweight or obese women improve fertility treatment outcomes? A systematic review. Obes. Rev..

[140] Garvey W.T., Batterham R.L., Bhatta M., Buscemi S., Christensen L.N., Frias J.P., Jódar E., Kandler K., Rigas G., Wadden T.A. (2022). Two-year effects of semaglutide in adults with overweight or obesity: The STEP 5 trial. Nat. Med..

